# Supplementary material for: Regorafenib inhibits EphA2 phosphorylation and leads to liver damage via the ERK/MDM2/p53 axis
Source: Nat Commun. 2023 May 13;14:2756. doi: 10.1038/s41467-023-38430-8 (PMC10182995; doi:10.1038/s41467-023-38430-8)
Supplement: Supplementary file 1 — Supplementary Information [file 41467_2023_38430_MOESM1_ESM.pdf]

Supplementary Information for:

Regorafenib inhibits EphA2 phosphorylation and leads to liver damage via the ERK/MDM2/p53 axis

Hao Yan [1], Wentong Wu [1], Yuhuai Hu [2,3], Jinjin Li [1], Jiangxin Xu [1], Xueqin Chen [4,5], Zhifei Xu [1], Xiaochun Yang [1], Bo Yang [6], Qiaojun He [1,2], Peihua Luo [1,7,8*]

[1]Center for Drug Safety Evaluation and Research of Zhejiang University, College of Pharmaceutical Sciences, Zhejiang University, Hangzhou 310058, China

[2]Innovation Institute for Artificial Intelligence in Medicine of Zhejiang University, Hangzhou 310018, China

[3]Laboratory of Fruit Quality Biology/Zhejiang Provincial Key Laboratory of Horticultural Plant Integrative Biology/The State Agriculture Ministry Laboratory of Horticultural Plant Growth, Development and Quality Improvement, Zhejiang University, Hangzhou, 310058, China

[4]Department of Oncology, Affiliated Hangzhou Cancer Hospital, Zhejiang University School of Medicine, Key Laboratory of Clinical Cancer Pharmacology and Toxicology Research of Zhejiang Province, Hangzhou 310002, China

[5]Cancer Center, Zhejiang University, Hangzhou 310058, China

[6]Institute of Pharmacology & Toxicology, College of Pharmaceutical Sciences, Zhejiang University, Hangzhou 310058, China

[7]Department of Pharmacology and Toxicology, Hangzhou Institute of Innovative Medicine, College of Pharmaceutical Sciences, Zhejiang University, Hangzhou, 310018, China

[8]Key Laboratory of Clinical Cancer Pharmacology and Toxicology Research of Zhejiang Province, Affiliated Hangzhou Cancer Hospital, Zhejiang University School of Medicine, Hangzhou, 310002, China

Supplementary information includes 23 Supplementary figures and 5 Supplementary tables.

Supplementary figures and legends

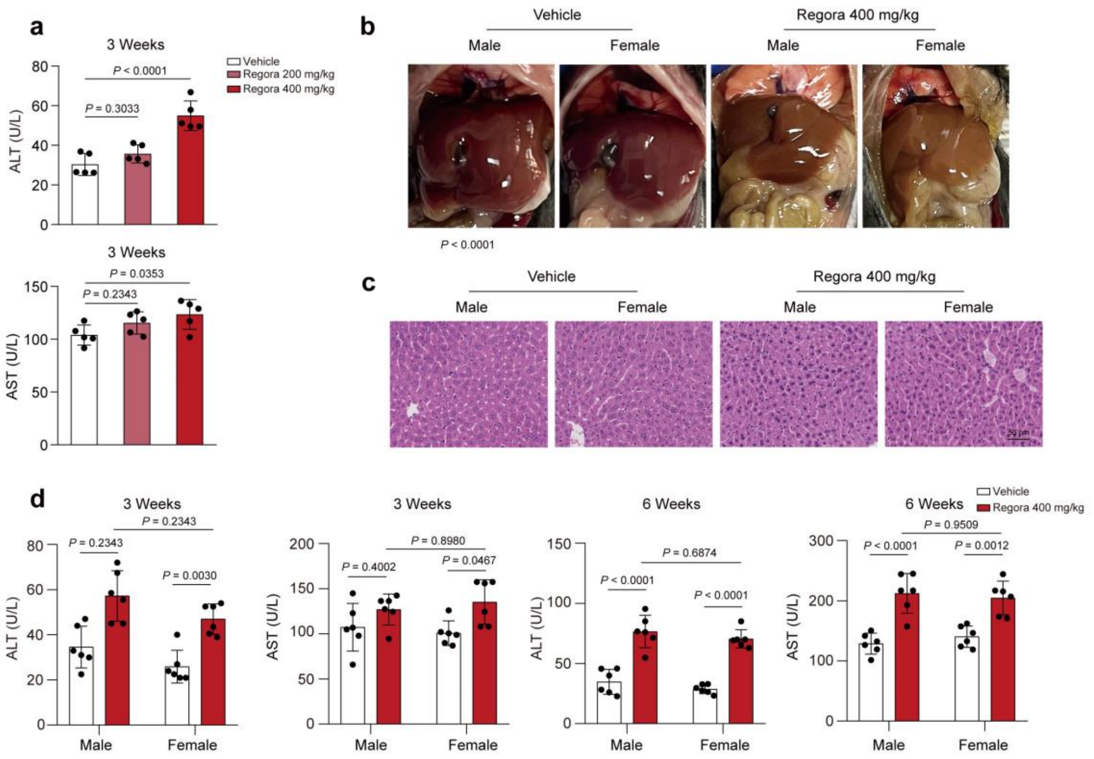

Supplementary Figure 1. Regorafenib caused liver injury with different dosage, duration and sexes *in vivo.* **a** C57BL/6J male mice were treated with 0.5% CMC-Na, 200 mg/kg/day regorafenib or 400 mg/kg/day regorafenib for 3 weeks. The levels of serum ALT and AST were analysed (n = 5 per group). **b-d** C57BL/6J mice including male and female were treated with 0.5% CMC-Na or 400 mg/kg/day regorafenib for 6 weeks (n = 6 per group). **b** Representative images of liver. **c** Representative images of H&E staining. scale bar: 50 μm. **d** The levels of serum ALT and AST when C57BL/6J mice were administered for 3 or 6 weeks were analysed (n = 6 per group). Data were expressed as mean ± SD. One way ANOVA followed by Tukey post hoc test for (**a** and **d**). Source data are provided as a Source Data file. Regora, regorafenib.

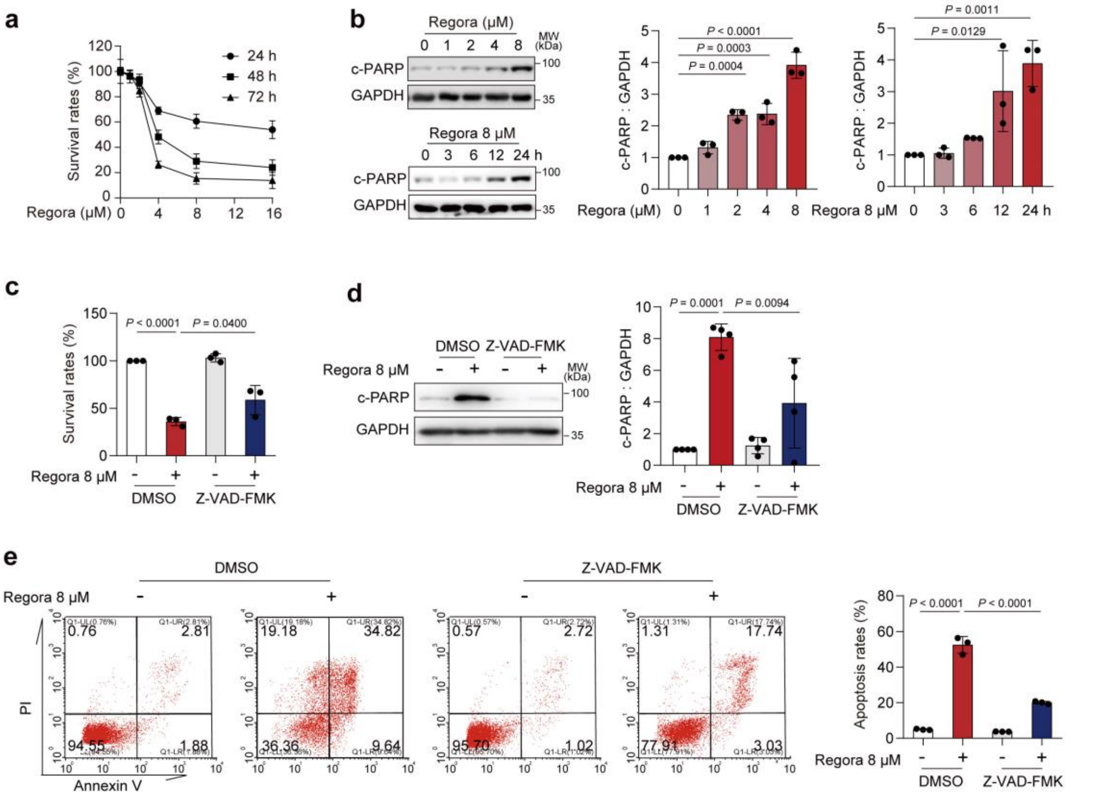

Supplementary Figure 2. Regorafenib induced hepatocytes apoptosis. a The survival rates of HL-7702 cells treated with 0, 4, 8, 12 or 16 μM regorafenib for 24 h, 48 h or 72 h were detected by SRB staining. *n* = 3 independent experiments. **b** HL-7702 cells were treated with 0, 1, 2, 4 or 8 μM regorafenib for 24 h (upper) or treated with 8 μM regorafenib for 0, 3, 6, 12 or 24 h (lower). The expression level of c-PARP was measured by western blot. *n* = 3 independent experiments. **c-e** HL-7702 cells were treated with DMSO or 8 μM regorafenib and/or 20 μM Z-VAD-FMK for 48 h (for survival rate and apoptosis rate measurement) or 24 h (for protein expression level measurement). *n* = 3 independent experiments for (**c** and **e**) and *n* = 4 independent experiments for (**d**). **c** The survival rates of HL-7702 cells were detected by SRB staining. **d** The expression level of c-PARP was detected by western blot. **e** The apoptosis rates were measured by flow cytometry analysis with Annexin V-PI staining. Data were expressed as mean ± SD. One way ANOVA followed by Tukey post hoc test for (**b-e**). Source data are provided as a

Source Data file. Regora, regorafenib; MW, molecular weight.

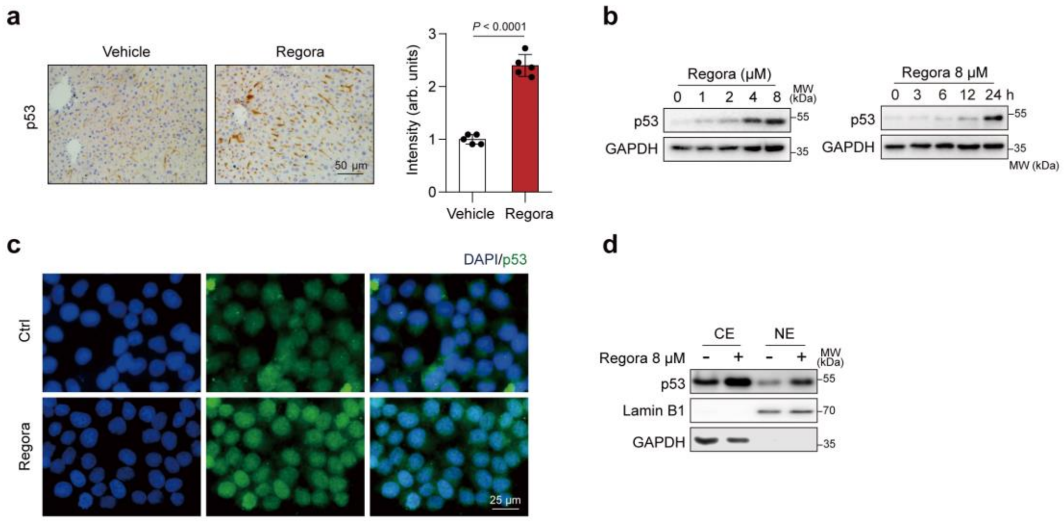

Supplementary Figure 3. Regorafenib induced upregulation of p53 in hepatocytes. **a** Representative image of immunohistochemistry staining for p53 in liver tissues treated with 0.5% CMC-Na or 400 mg/kg/day regorafenib for 6 weeks. Scale bar: 50 μm. The immunohistochemistry for p53 staining was quantified by densitometric analysis. One area from five mice per group. **b** HL-7702 cells were treated with 0, 1, 2, 4 or 8 μM regorafenib for 24 h or treated with 8 μM regorafenib for 0, 3, 6, 12 or 24 h. The expression level of p53 was detected by western blot. Blots are representative of three independent experiments. **c** Representative images of immunofluorescence for p53 (green) in HL-7702 cells treated with or without 8 μM regorafenib for 24 h from three independent experiments. Scale bar: 25 μm. **d** HL-7702 cells were treated with 8 μM regorafenib for 24 h. The expression level of p53 in cytoplasm and nuclei was detected by western blot. Blots are representative of two independent experiments. Data were expressed as mean ± SD. Unpaired two-sided student's *t*-test for (**a**). Source data are provided as a Source Data file. Regora, regorafenib; Ctrl, control; CE, cytoplasmic extraction; NE, nuclear extraction; MW, molecular weight.

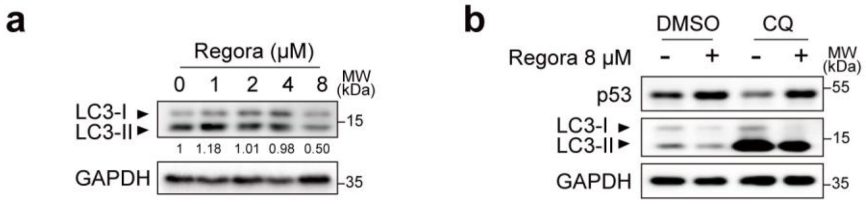

Supplementary Figure 4. The elevation of p53 caused by regorafenib is not related to autophagic degradation. a HL-7702 cells were treated with 0, 1, 2, 4 or 8 μM regorafenib for 24 h. The expression level of LC3 was detected by western blot. Blots are representative of two independent experiments. **b** HL-7702 cells were treated with or without 8 μM regorafenib and/or 10 μM CQ for 24 h. The expression levels of p53 and LC3 were detected by western blot. Blots are representative of two independent experiments. Source data are provided as a Source Data file. Regora, regorafenib; CQ, chloroquine; MW, molecular weight.

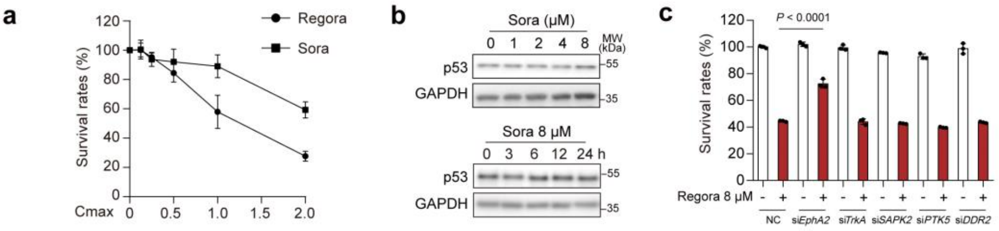

Supplementary Figure 5. EphA2 is a key target for regorafenib to induce hepatotoxicity. a The survival rates of HL-7702 cells treated with 0, 0.5, 1.0, 1.5 or 2.0 times Cmax of regorafenib or sorafenib for 24 h were detected by SRB staining. *n* = 3 independent experiments. **b** HL-7702 cells were treated with 0, 1, 2, 4 or 8 μM sorafenib for 24 h or treated with 8 μM sorafenib for 0, 3, 6, 12 or 24 h. The expression level of p53 was detected by western blot. Blots are representative of three independent experiments. **c** HL-7702 cells were transfected with si*EpHA2*, si*TrkA*, si*SAPK2*, si*PTK5* or si*DDR2* for 24 h before treating with 8 μM regorafenib for 48 h. The survival rates were detected by SRB staining. *n* = 3 independent experiments. Data were expressed as mean ± SD. One way ANOVA followed by Tukey post hoc test for **c**. Source data are provided as a Source Data file. Regora, regorafenib; Sora, sorafenib; MW, molecular weight.

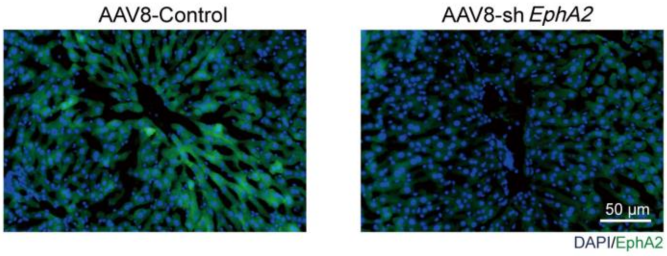

Supplementary Figure 6. The efficiency of EphA2 knockdown in mouse liver. AAV8-TBG-Control or AAV8-TBG-sh*EphA2* were injected into C57BL/6J mice for 6 weeks through tail vein. The knockdown efficiency of EphA2 (green) was detected by immunofluorescence analysis. Representative image from three mice liver section per group. Scale bar: 50 μm.

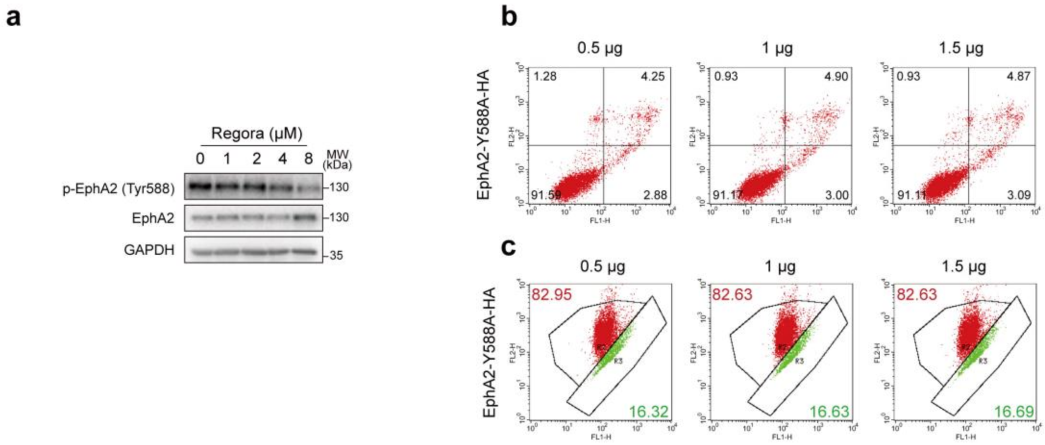

Supplementary Figure 7. Inhibition of p-EphA2 (Tyr588) is not associated with regorafenib-induced cell apoptosis. a The expression levels of p-EphA2 (Tyr588) and EphA2 in HL-7702 cells treated with 0, 1, 2, 4 or 8 μM regorafenib for 24 h were analysed by western blot. Blots are representative of two independent experiments. **b-c** HL-7702 cells were transfected with 0.5, 1 or 1.5 μg EphA2 Y588A plasmid for 48 h (for apoptosis rate measurement) or 24 h (for MMP measurement). **b** Representative images of cell apoptosis rates measured by flow cytometry with Annexin V-PI staining (*n* = 2 independent experiments). **c** Representative images of mitochondrial membrane potential measured by flow cytometry with JC-1 staining (*n* = 2 independent experiments). Source data are provided as a Source Data file. Regora, regorafenib; MW, molecular weight.

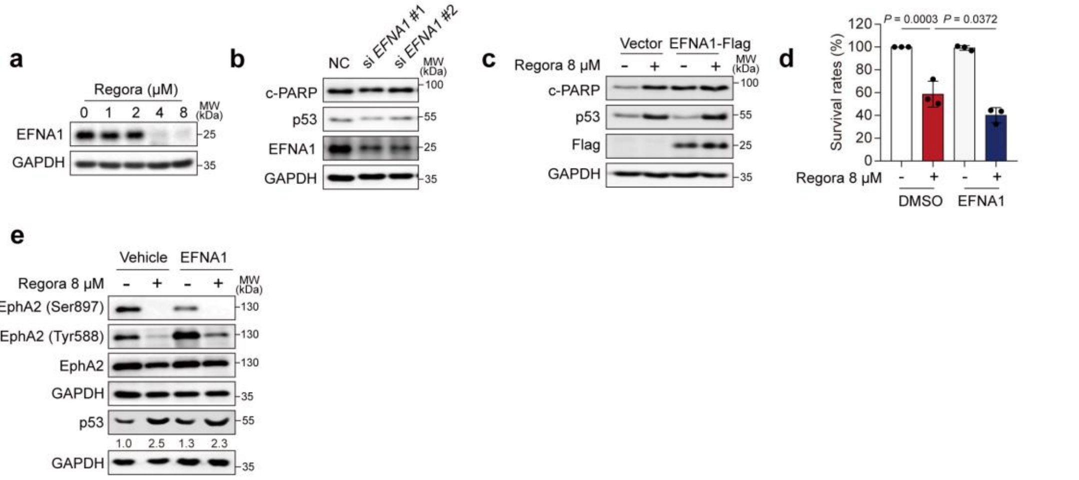

Supplementary Figure 8. The EphA2 ligand EFNA1 mediated canonical sigalling pathway is not involved in regorafenib-induced hepatotoxicity. a The expression levels of EFNA1 in HL-7702 cells treated with 0, 1, 2, 4 or 8 μM regorafenib for 24 h were analysed by western blot. **b** HL-7702 cells were incubated with negative non-targeting siRNA (NC) or EFNA1 siRNA (si*EFNA1* #1, si*EFNA1* #2), followed by treatment with or without 8 μM regorafenib for 24 h. The expression levels of c-PARP, p53 and EFNA1 were analysed by western blot. **c** HL-7702 cells were transfected with 1 μg vector or EFNA1 plasmid, followed by treatment with or without 8 μM regorafenib for 24 h. The expression levels of c-PARP, p53 and Flag were analysed by western blot. **d-e** HL-7702 cells were treated with 100 ng/mL EFNA1 or 8 μM regorafenib for 36 h (for survival rate measurement) or 24 h (for western blot analysis). **d** SRB staining analysis was carried out to determine the survival rates of HL-7702 cells. n = 3 independent experiments. **e** The expression levels of p-EphA2 (Ser897), p-EphA2 (Tyr588), EphA2 and p53 were analysed by western blot. Blots are representative of two independent experiments for (**a-c** and **e**). Data were expressed as mean ± SD. One way ANOVA followed by Tukey post hoc test for **d**. Source data are provided as a Source Data file. Regora, regorafenib; NC, negative control; MW, molecular weight.

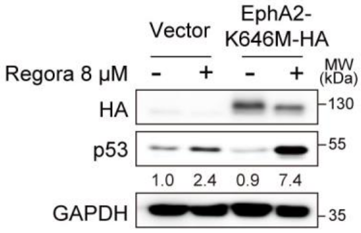

Supplementary Figure 9. Regorafenib increased the level of p53 independent on tyrosine kinase activity of EphA2. HL-7702 cells were transfected with 1 μg vector or EphA2-K646M plasmid, followed by treatment with or without 8 μM regorafenib for 24 h. The expression levels of HA and p53 were analysed by western blot. Blots are representative of two independent experiments. Source data are provided as a Source Data file. Regora, regorafenib; MW, molecular weight.

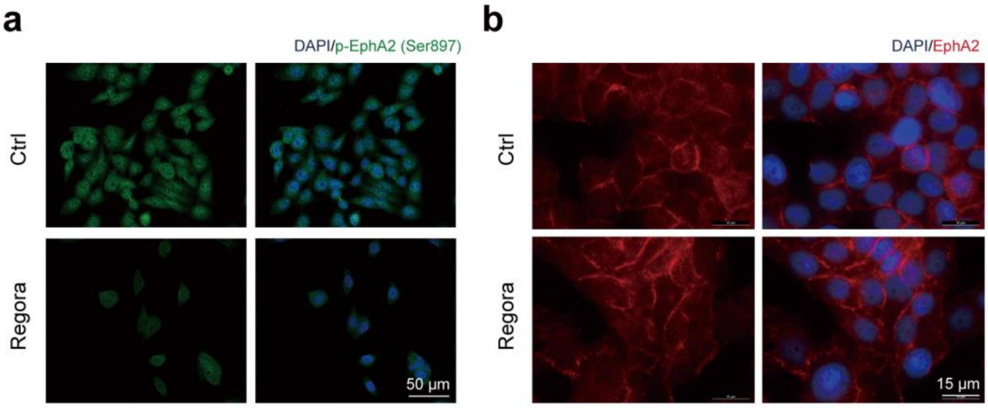

Supplementary Figure 10. p-EphA2 (Ser897) was inhibited by regorafenib. a HL-7702 cells were treated with 8 μM regorafenib for 24 h. The intracellular distribution and expression of p-EphA2 (Ser897) (green) was observed by immunofluorescence. Scale bar: 50 μm. **b** Immunofluorescence analysis of EphA2 (red) in HL-7702 cells after treatment of 8 μM regorafenib for 24 h. Representative images are shown from three independent experiments for (**a** and **b**). Scale bar: 15 μm. Ctrl, control; Regora, regorafenib.

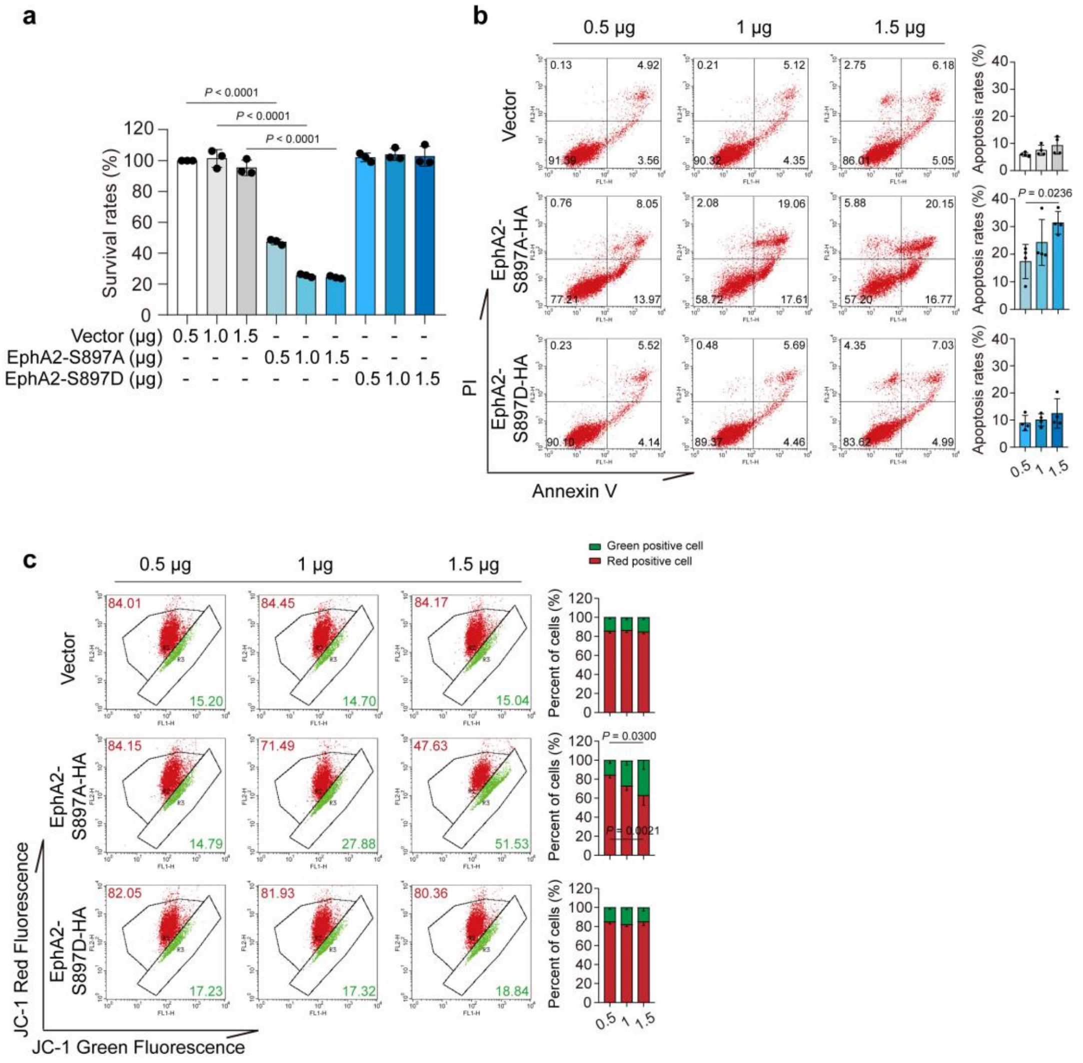

Supplementary Figure 11. Inhibition of p-EphA2 (Ser897) is related to hepatocyte apoptosis and mitochondrial dysfunction. a HL-7702 cells were transfected with 0.5, 1.0 or 1.5 μg vector, EphA2 S897A plasmid or EphA2 S897D plasmid for 48 h. The survival rates of HL-7702 cells were measured by SRB staining. *n* = 3 independent experiments. **b-c** HL-7702 cells were transfected with 0.5, 1.0 or 1.5 μg EphA2 S897A plasmid or EphA2 S897D plasmid for 48 h (for apoptosis rate measurement) or 24 h (for MMP measurement). **b** The apoptosis rates were measured by flow cytometry analysis of Annexin V-PI staining. *n* = 4 independent experiments. **c** MMP was detected by JC-1 staining and flow cytometry. *n* = 3 independent experiments. Data were expressed as

mean ± SD. One way ANOVA followed by Tukey post hoc test for (**a**-**c**). Source data are provided as a Source Data file.

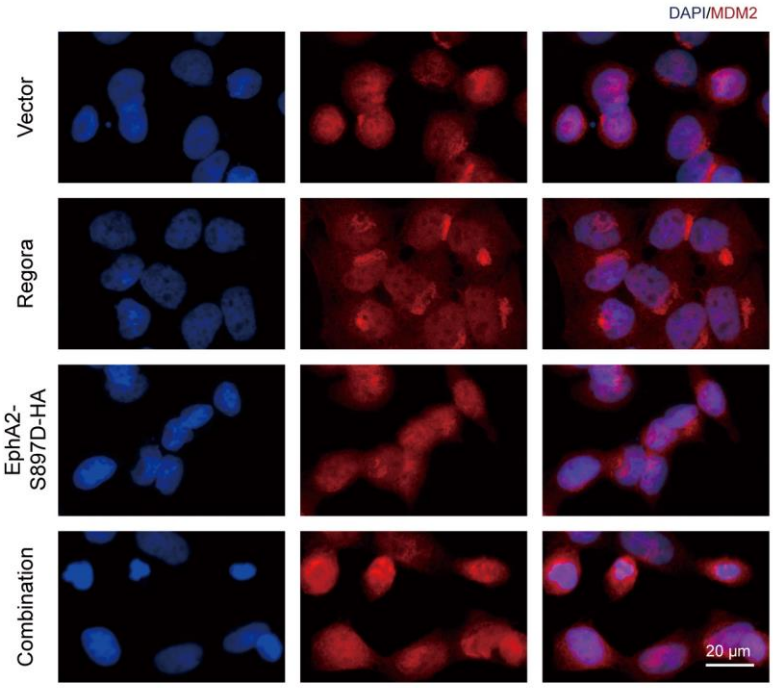

Supplementary Figure 12. Regorafenib changed the distribution of MDM2 by inhibiting the phosphorylation of EphA2^{Ser897}. HL-7702 cells were treated with 8 μM regorafenib for 24 h after transfected with 1.0 μg EphA2 S897D plasmid for 24 h. The distribution of MDM2 (red) in HL-7702 cells was observed by immunofluorescence. Representative images are shown from three independent experiments. Scale bar: 20 μm. Regora, regorafenib.

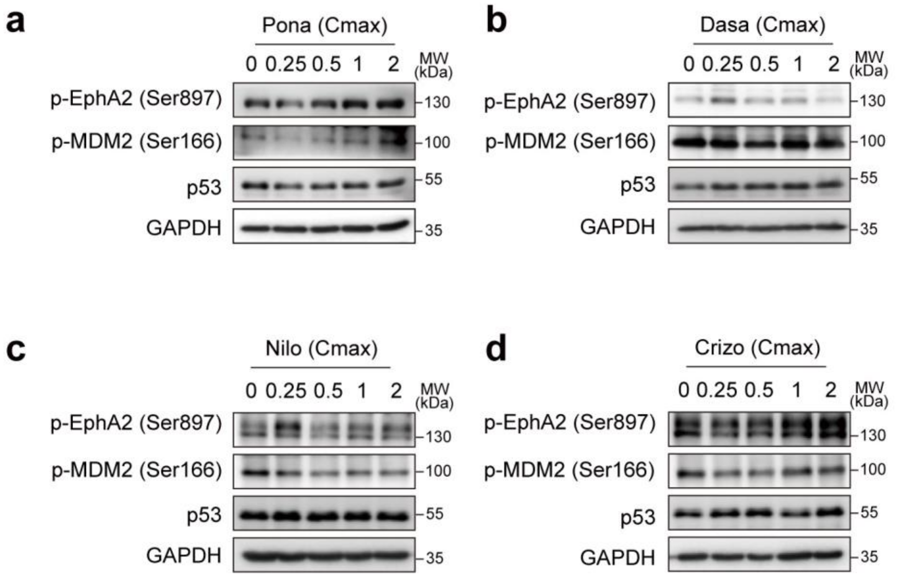

Supplementary Figure 13. The effect of EphA2 inhibitors on p-EphA2 (Ser897), p-MDM2 (Ser166) and p53. a-d HL-7702 cells were treated with 0, 0.25, 0.5, 1 or 2 multiples of Cmax of ponatinib, dasatinib, nilotinib, crizotinib for 24 h. The expression levels of p-EphA2 (Ser897), p-MDM2 (Ser166) and p53 were detected by western blot. Blots are representative of three independent experiments. Source data are provided as a Source Data file. Pona, ponatinib; Dasa, dasatinib; Nilo, nilotinib; Crizo, crizotinib; MW, molecular weight.

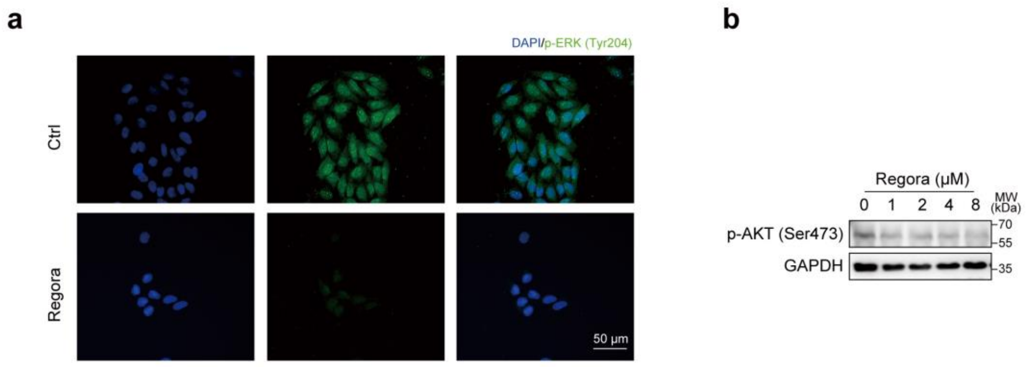

Supplementary Figure 14. p-ERK but not p-AKT was changed with the regorafenib treatment. a The distribution and expression of p-ERK (Tyr204) (green) in HL-7702 cells treated with 8 μM regorafenib were observed by immunofluorescence Representative images are shown from three independent experiments. Scale bar: 50 μm. **b** The expression levels of p-AKT (Ser473) in HL-7702 cells treated with 0, 1, 2, 4 or 8 μM regorafenib for 24 h were detected by western blot. Blots are representative of two independent experiments. Source data are provided as a Source Data file. Ctrl, control; Regora, regorafenib; MW, molecular might.

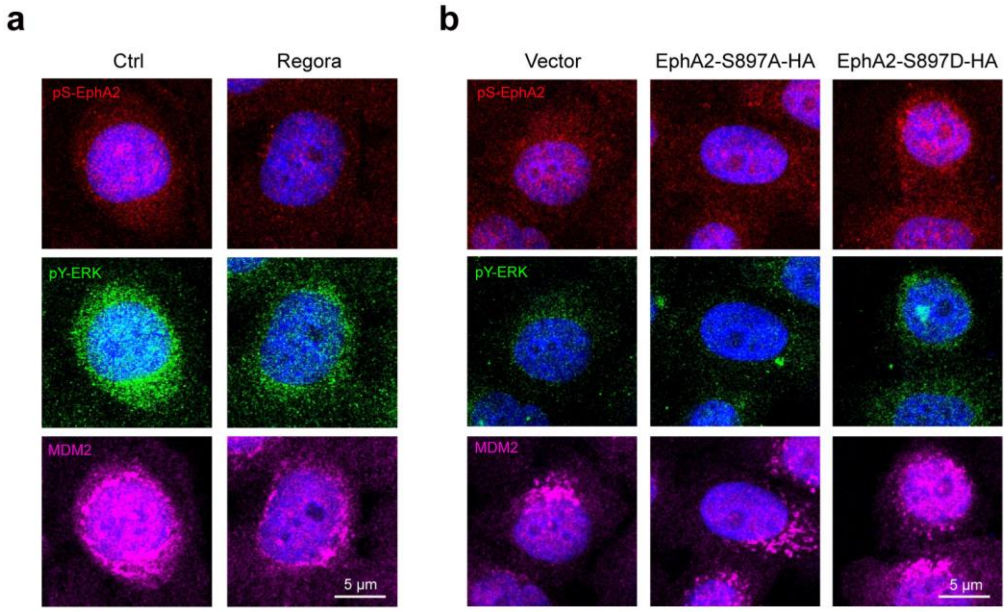

Supplementary Figure 15. p-EphA2 (Ser897) inhibition was associated with reduced p-ERK and the cytoplasmic retention of MDM2. a The expression levels of p-EphA2 (Ser897) (red), p-ERK (Tyr204) (green) and MDM2 (meganta) in HL-7702 cells treated with 8 μM regorafenib for 24 h were observed by immunofluorescence. Scale bar: 5 μm. **b** HL-7702 cells were transfected with 1.0 μg EphA2 S897A plasmid or EphA2 S897D plasmid for 24 h. The expression levels and localizations of p-EphA2 (Ser897) (red), p-ERK (Tyr204) (green) and MDM2 (meganta) were observed by immunofluorescence. Representative images are shown from three independent experiments for (**a** and **b**). Scale bar: 5 μm. Ctrl, control; Regora, regorafenib.

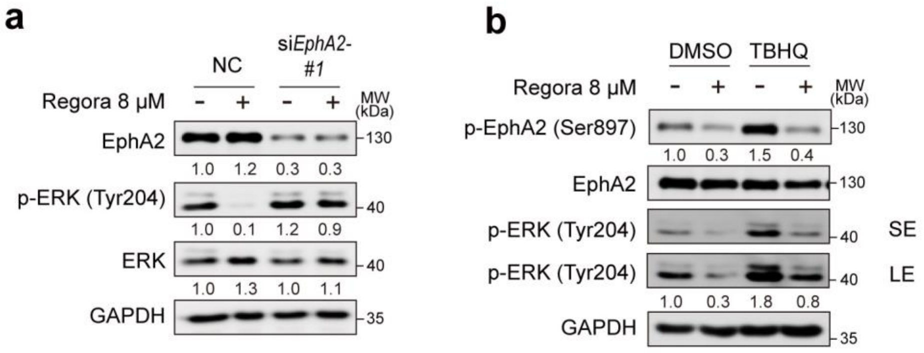

Supplementary Figure 16. EphA2 regulated ERK under regorafenib treatment. a HL-7702 cells were transfected with non-targeting siRNA (NC) or targeting EphA2 siRNA (si*EphA2* #1), followed by treatment with or without 8 μM regorafenib for 24 h). The expression levels of EphA2, p-ERK (Tyr204) and ERK in HL-7702 cells were detected by western blot. **b** HL-7702 cells were treated with 20 μM TBHQ and/or 8 μM regorafenib for 24 h. The expression levels of p-EphA2 (Ser897), EphA2 and p-ERK (Tyr204) were detected by western blot. Blots are representative of two independent experiments. Source data are provided as a Source Data file. Regora, regorafenib; NC, negative control; MW, molecular weight; SE, short exposure; LE, long exposure.

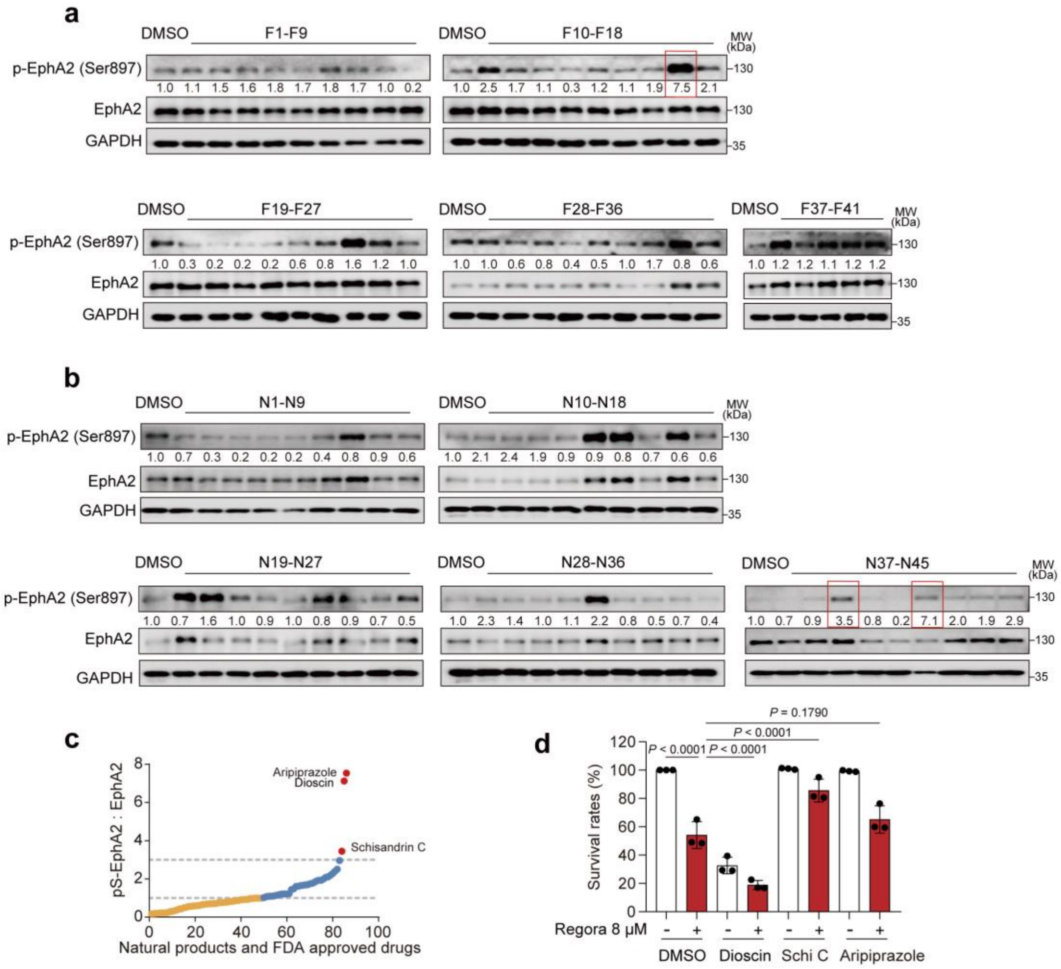

Supplementary Figure 17. Drug screening for the intervention of regorafenib-induced hepatotoxicity. a The expression levels of p-EphA2 (Ser897) and EphA2 in HL-7702 cells treated with 10 μM FDA approved drugs (n = 1). **b** The expression levels of p-EphA2 (Ser897) and EphA2 in HL-7702 cells treated with 40 μM natural products (n = 1). **c** The scatter diagram of fold change of pS-EphA2:EphA2 in HL-7702 cells. **d** The survival rates were detected when combined regorafenib with aripiprazole, schisandrin C and dioscin for 36 h. n = 3 independent experiments. Data were expressed as mean ± SD. One way ANOVA followed by Tukey post hoc test for d. Source data are provided as a Source Data file. Regora, regorafenib; Schi C, schisandrin C.

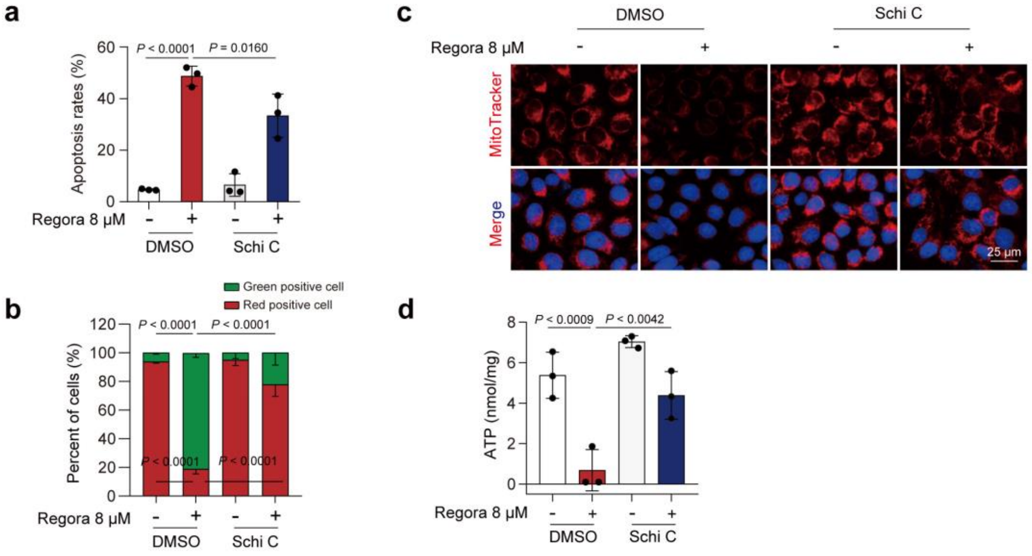

Supplementary Figure 18. Schisandrin C effectively alleviated regorafenib-induced apoptosis and mitochondrial dysfunction. a-d HL7702 cells were treated with 8 μM regorafenib and/or 40 μM schisandrin C for 48 h (for apoptosis analysis) or 24 h (for JC-1 staining, mitochondrial mass analysis and ATP detection). *n* = 3 independent experiments. **a** The apoptosis rates were measured by flow cytometry analysis with Annexin V-PI staining. **b** MMP was detected by flow cytometry with JC-1 staining. **c** The mitochondrial mass in HL-7702 cells was detected by MitoTracker staining. Representative images are shown. **d** The concentration of ATP in HL-7702 cells were measured by ATP detection kit. Data were expressed as mean ± SD. One way ANOVA followed by Tukey post hoc test for (**a**, **b** and **d**). Source data are provided as a Source Data file. Regora, regorafenib; Schi C, schisandrin C.

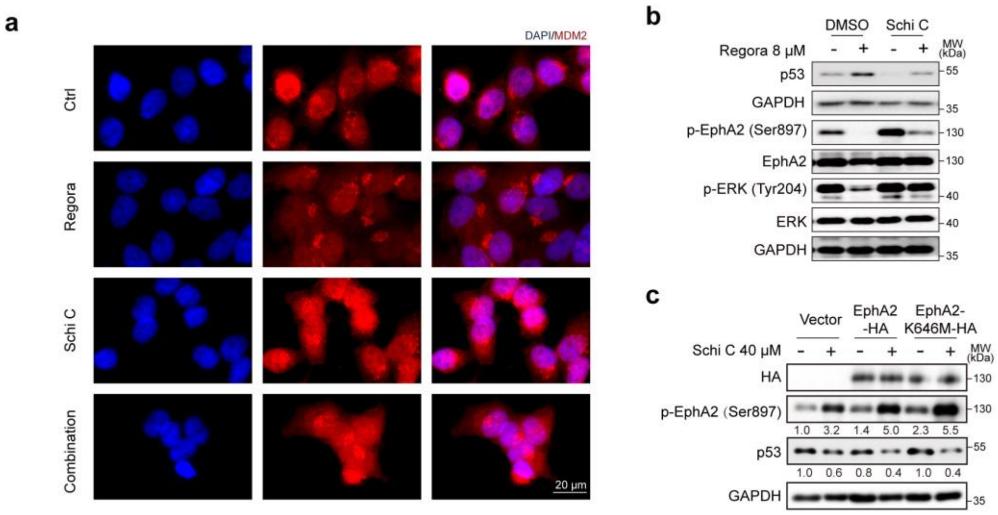

Supplementary Figure 19. Schisandrin C improved regorafenib-induced MDM2 cytoplasmic retention and increased p53 by upregulating p-EphA2 (Ser897). a, b HL-7702 cells were treated with 8 μM regorafenib and/or 40 μM schisandrin C for 24 h. **a** Representative images of immunofluorescence for MDM2 (red) in HL-7702 cells from three independent experiments. Scale bar: 20 μm. **b** The expression levels of p-EphA2 (Ser897), p-EKR (Tyr204) and p53 in HL-7702 cells were detected by western blot. Blots are representative of three independent experiments. **c** HL-7702 cells were transfected with 1 μg vector EphA2 or EphA2-K646M plasmid, followed by treatment with or without 8 μM regorafenib for 24 h. The expression levels of HA, p-EphA2 (Ser897) and p53 were analysed by western blot. Blots are representative of two independent experiments. Source data are provided as a Source Data file. Ctrl, control; Regora, regorafenib; Schi C, schisandrin C; MW, molecular weight.

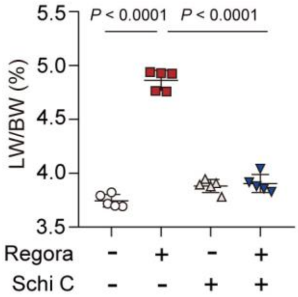

Supplementary Figure 20. Schizandra C ameliorated regorafenib-induced upregulation of LW/BW. The level of LW/BW in mice treated with 400 mg/kg/day regorafenib and/or 10 mg/kg/day schisandrin C for 6 weeks was detected (n = 5 per group). Data were expressed as mean ± SD. One way ANOVA followed by Tukey post hoc test. Source data are provided as a Source Data file. Regora, regorafenib; Schi C, schisandrin C; LW, liver weight; BW, body weight.

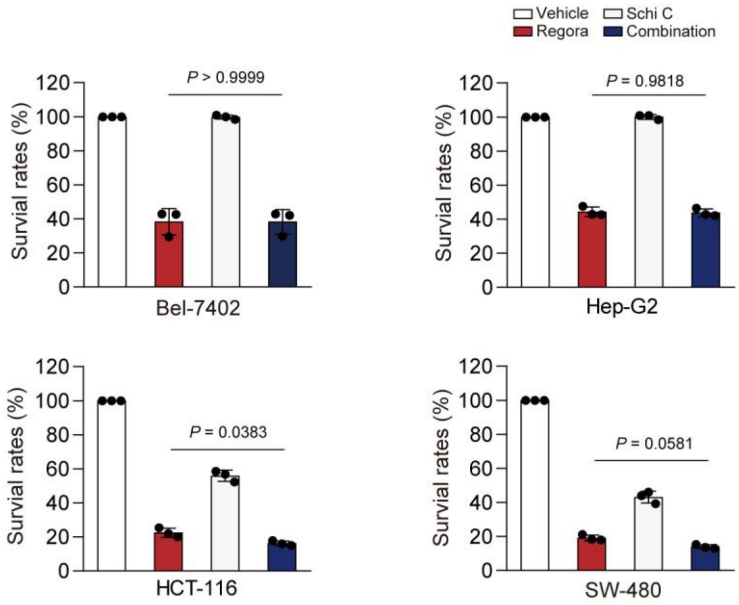

Supplementary Figure 21. Schizandra C didn't affect the anti-tumor effect of regorafenib. Two hepatocellular carcinoma cell lines (Bel-7402 and Hep-G2) and two colon cancer cell lines (HCT-116 and SW-480) were treated with or without 8 μM regorafenib and/or 40 μM schisandrin C for 48 h. SRB staining analysis was carried out to determine the survival rates. $n = 3$ independent experiments. Data were expressed as mean ± SD. One way ANOVA followed by Tukey post hoc test. Source data are provided as a Source Data file. Regora, regorafenib; Schi C, schisandrin C.

1. Full-length gels and blots of Supplementary Figure 2b (Page RulerTM Prestained NIR Protein Ladder: Product# 26616)

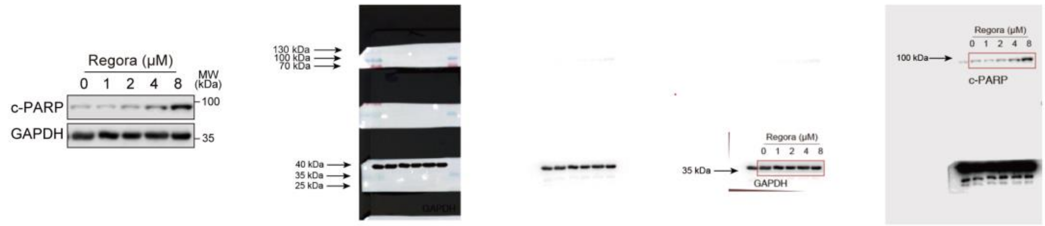

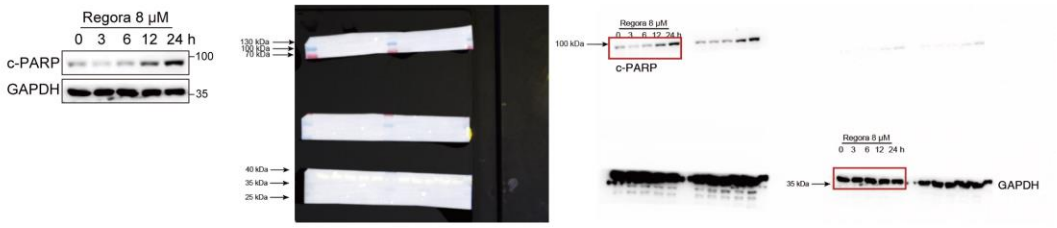

2. Full-length gels and blots of Supplementary Figure 2d (Page RulerTM Prestained NIR Protein Ladder: Product# 26616)

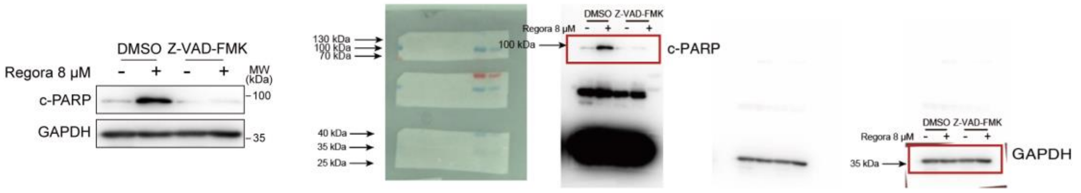

3. Full-length gels and blots of Supplementary Figure 3b (Page RulerTM Prestained NIR Protein Ladder: Product# 26616)

Supplementary figure 3b-left

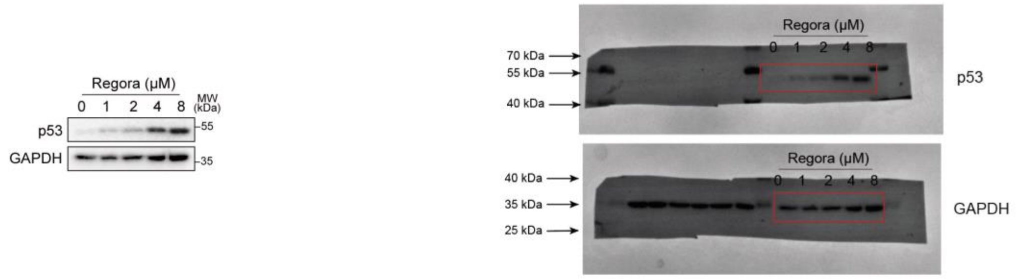

Supplementary figure 3b-right

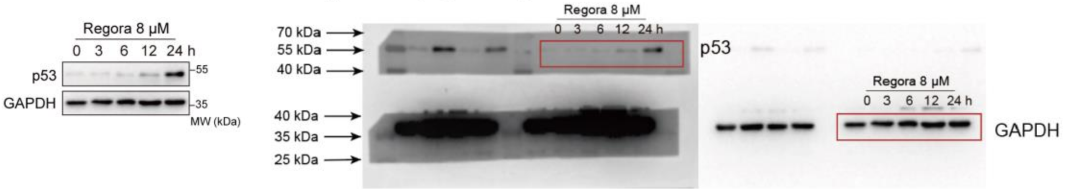

4. Full-length gels and blots of Supplementary Figure 3d (Page RulerTM Prestained NIR Protein Ladder: Product# 26616)

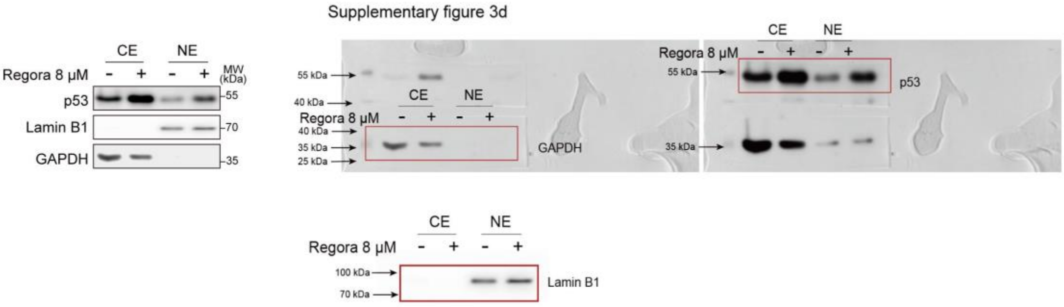

5. Full-length gels and blots of Supplementary Figure 4a (Page RulerTM Prestained NIR Protein Ladder: Product# 26616)

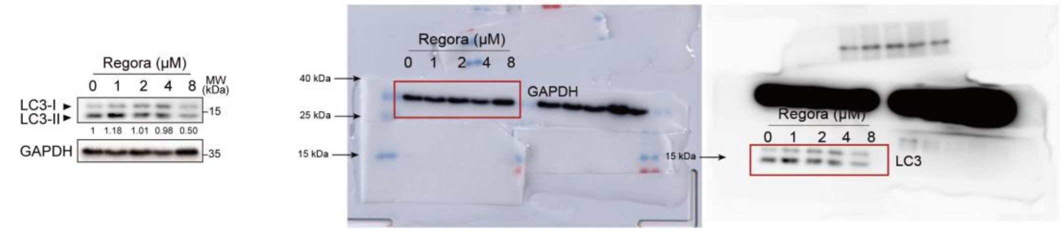

6. Full-length gels and blots of Supplementary Figure 4b (Page RulerTM Prestained NIR Protein Ladder: Product# 26616)

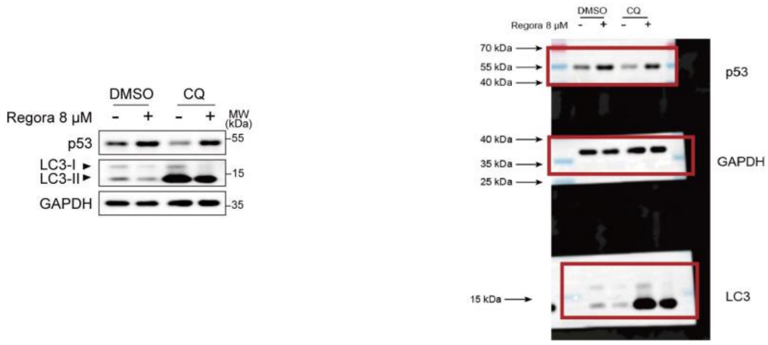

7. Full-length gels and blots of Supplementary Figure 5b (Page RulerTM Prestained NIR Protein Ladder: Product# 26616)

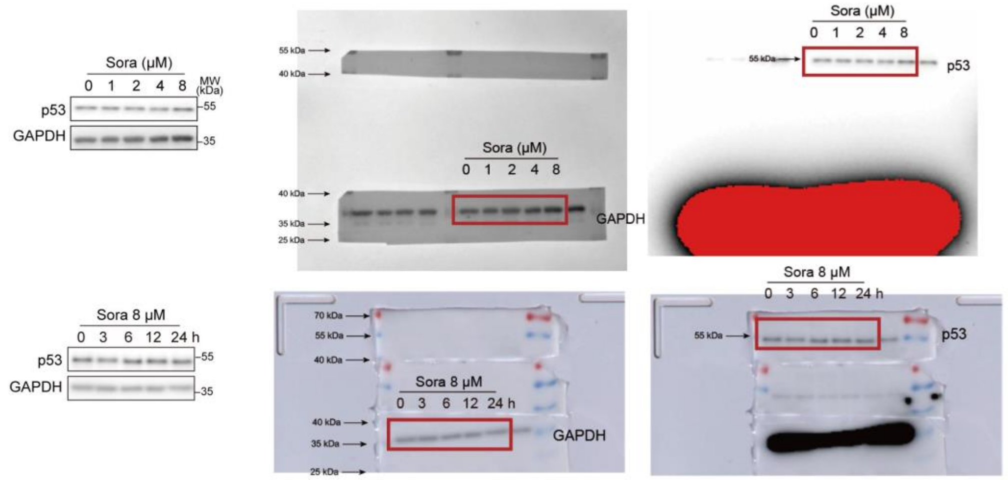

8. Full-length gels and blots of Supplementary Figure 7a (Page RulerTM Prestained NIR Protein Ladder: Product# 26616)

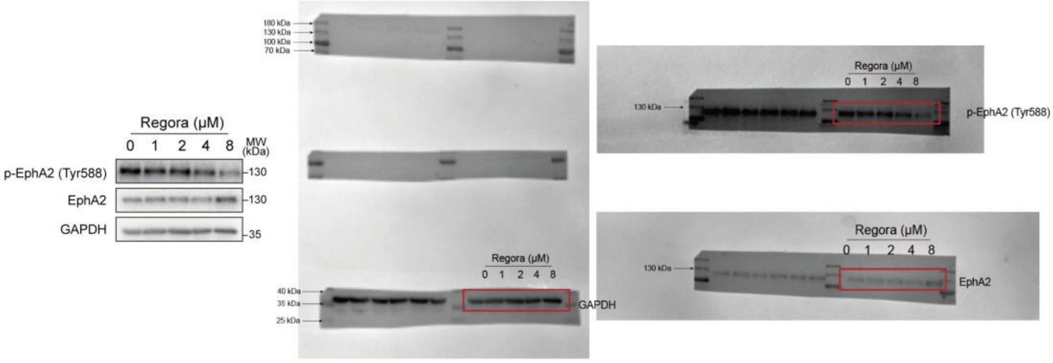

9. Full-length gels and blots of Supplementary Figure 8a (Page RulerTM Prestained NIR Protein Ladder: Product# 26616)

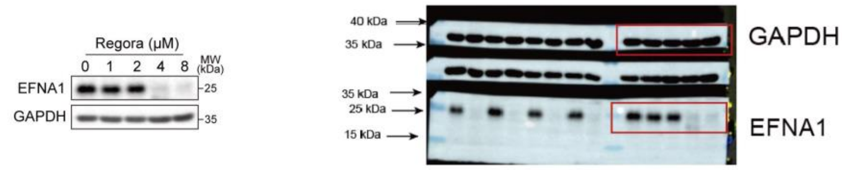

10. Full-length gels and blots of Supplementary Figure 8b (Page RulerTM Prestained NIR Protein Ladder: Product# 26616)

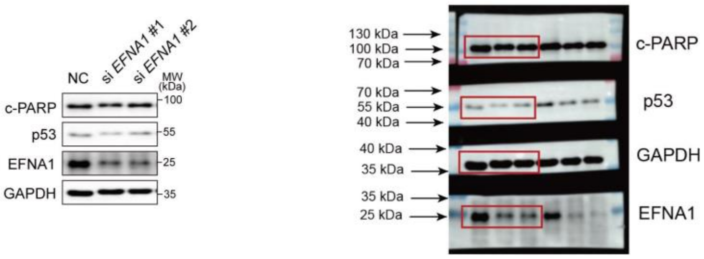

11. Full-length gels and blots of Supplementary Figure 8c (Page RulerTM Prestained NIR Protein Ladder: Product# 26616)

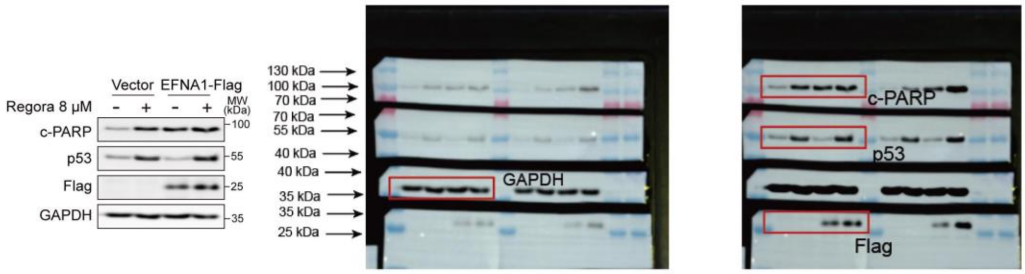

12. Full-length gels and blots of Supplementary Figure 8e (Page RulerTM Prestained NIR Protein Ladder: Product# 26616)

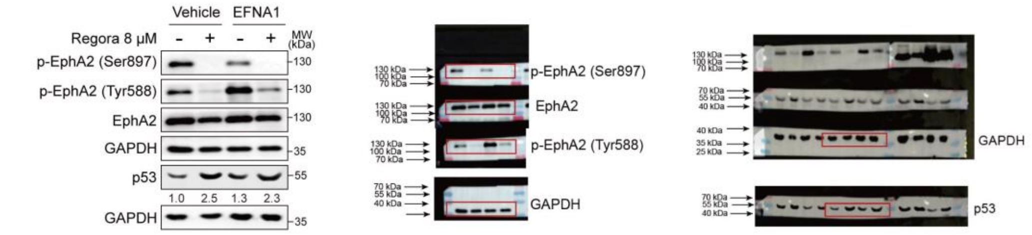

13. Full-length gels and blots of Supplementary Figure 9 (Page RulerTM Prestained NIR Protein Ladder: Product# 26616)

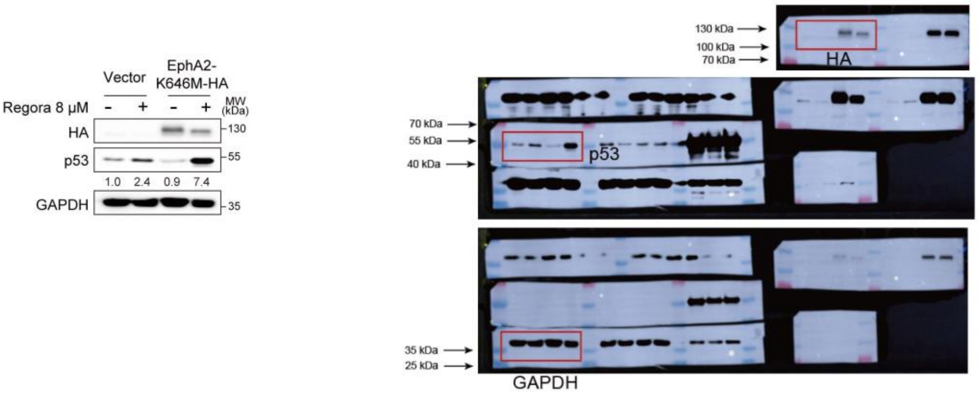

14. Full-length gels and blots of Supplementary Figure 13a (Page RulerTM Prestained NIR Protein Ladder: Product# 26616)

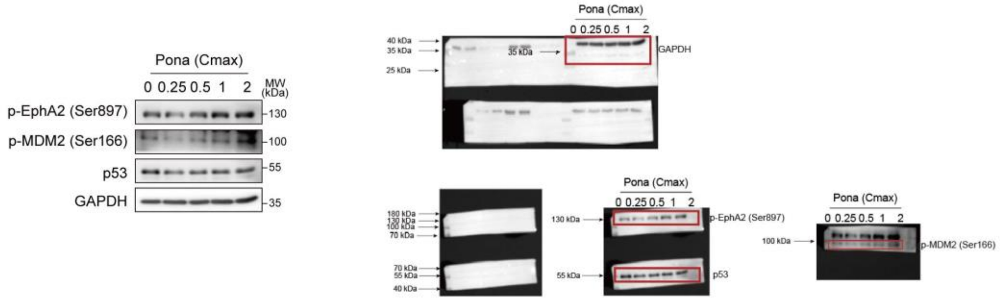

15. Full-length gels and blots of Supplementary Figure 13b (Page RulerTM Prestained NIR Protein Ladder: Product# 26616)

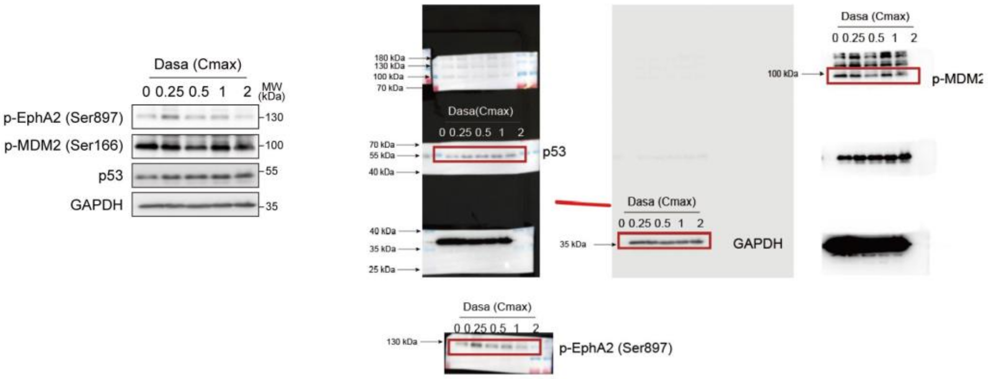

16. Full-length gels and blots of Supplementary Figure 13c and 13d (Page RulerTM Prestained NIR Protein Ladder: Product# 26616)

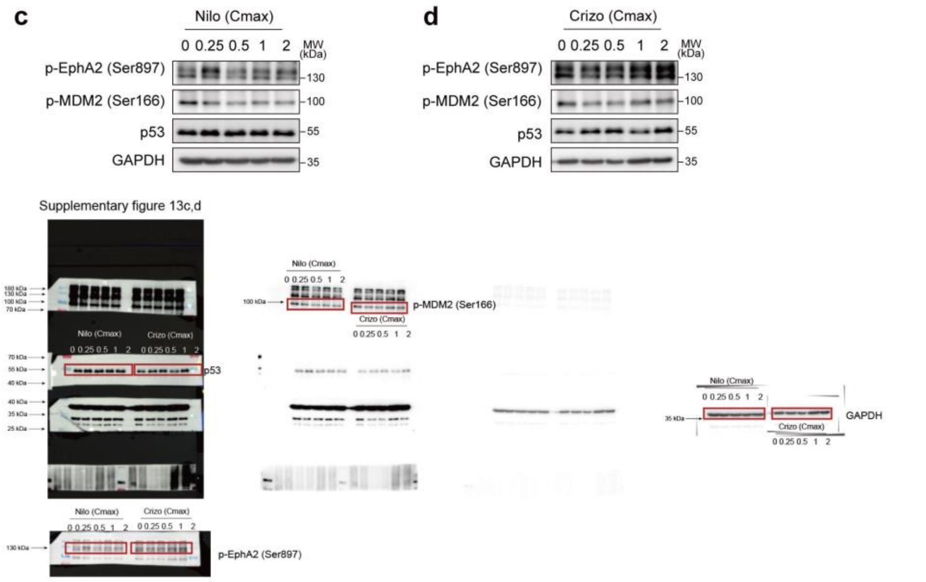

17. Full-length gels and blots of Supplementary Figure 14b (Page RulerTM Prestained NIR Protein Ladder: Product# 26616)

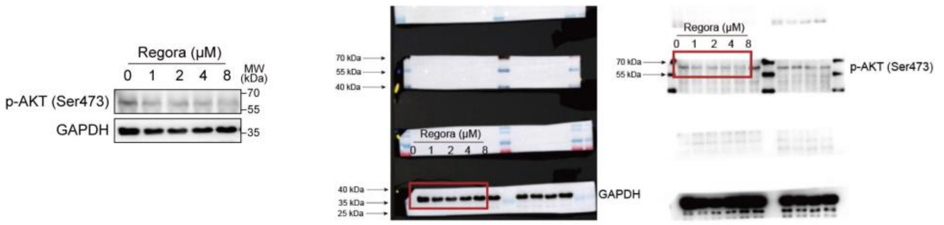

18. Full-length gels and blots of Supplementary Figure 16a (Page RulerTM Prestained NIR Protein Ladder: Product# 26616)

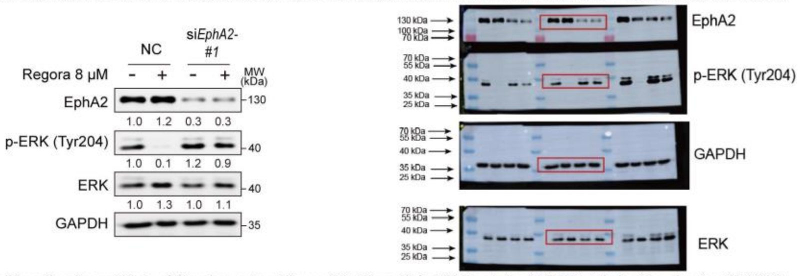

19. Full-length gels and blots of Supplementary Figure 16b (Page RulerTM Prestained NIR Protein Ladder: Product# 26616)

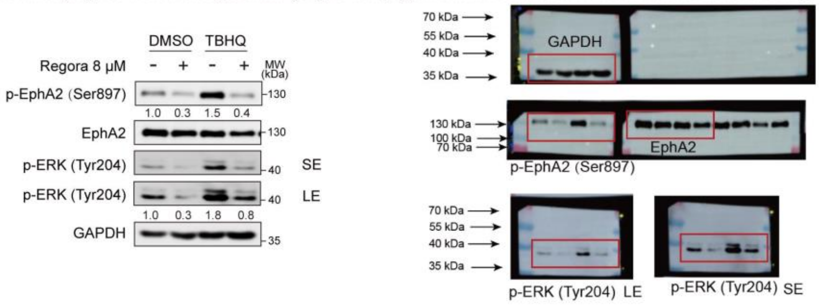

20. Full-length gels and blots of Supplementary Figure 17a (Page RulerTM Prestained NIR Protein Ladder: Product# 26616)

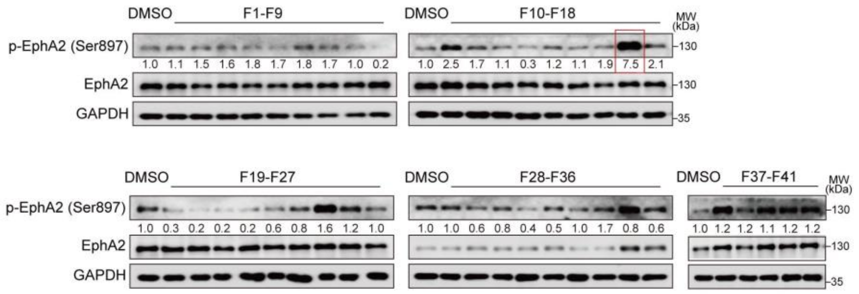

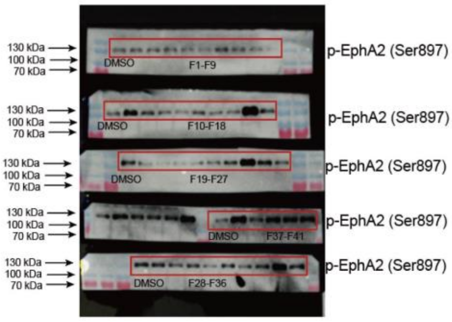

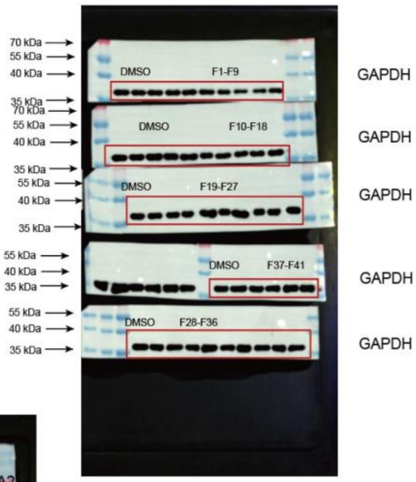

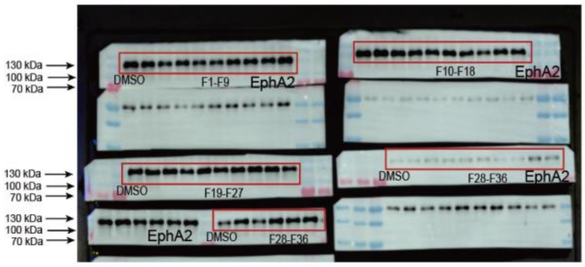

21. Full-length gels and blots of Supplementary Figure 17b (Page RulerTM Prestained NIR Protein Ladder: Product# 26616)

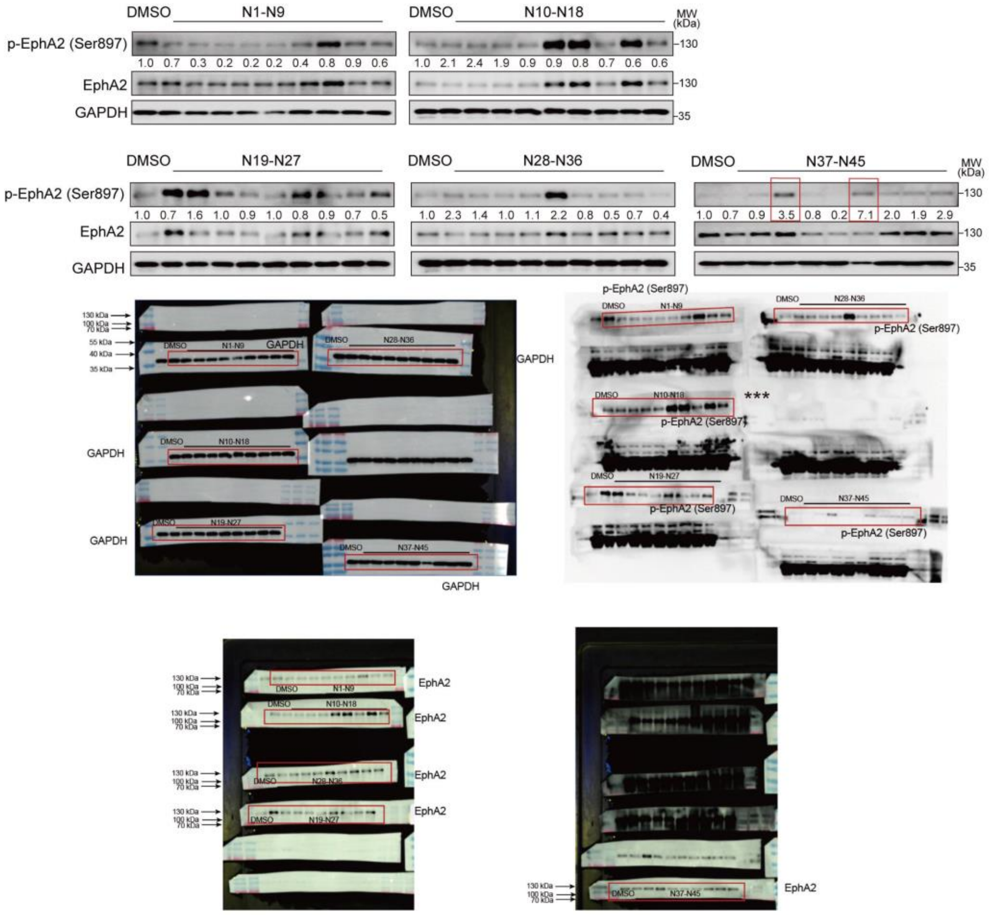

22. Full-length gels and blots of Supplementary Figure 19b (Page RulerTM Prestained NIR Protein Ladder: Product# 26616)

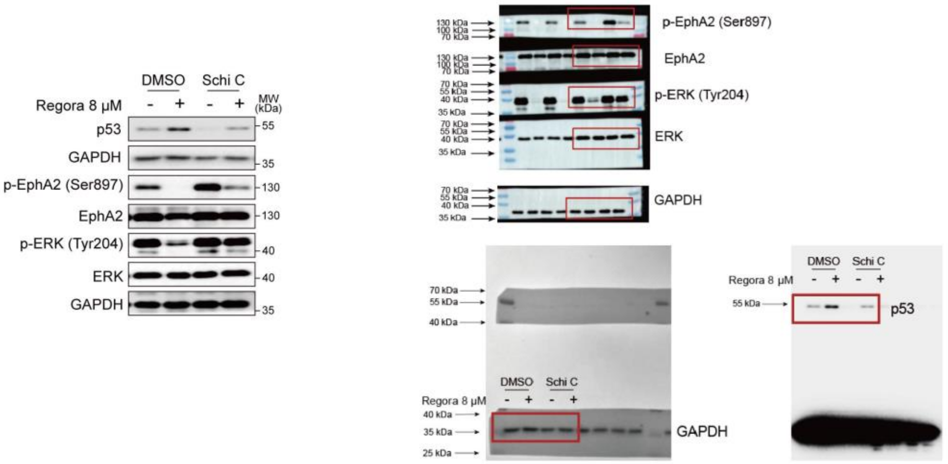

23. Full-length gels and blots of Supplementary Figure 19c (Page RulerTM Prestained NIR Protein Ladder: Product# 26616)

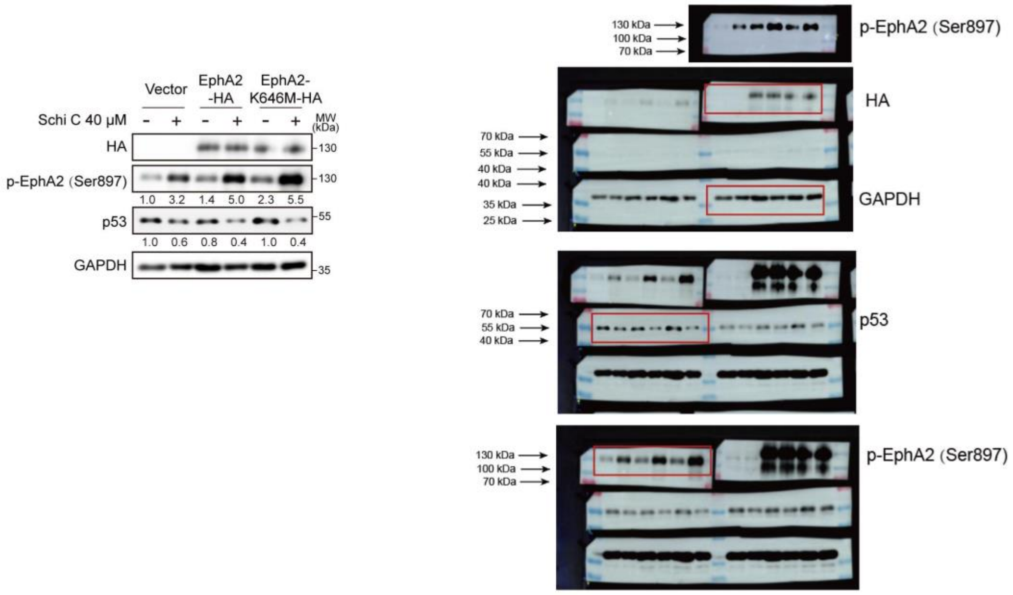

Supplementary Figure 22. The respective original western blot images. The western blot images presented in papers and their corresponding original images.

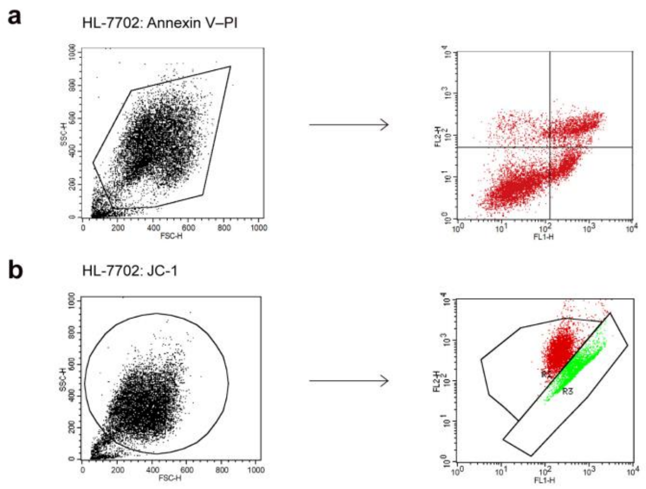

Supplementary Figure 23. Gating strategies of flow cytometry. a Gating strategy to determine the percentage of Annexin V-FITC^{+}PI^{+} and Annexin V-FITC^{+}-PI^{-} cells presented in Supplementary Fig. 11b. **b** Gating strategy to determine the percentage of JC-1 staning Red cells and Green cells presented in Supplementary Fig. 11c.

Supplementary tables

Supplementary Table 1. Characteristics of the human primary hepatocytes

Donor	HPH-1	HPH-2	HPH-3
Product	M00995-P	M00995-P	F00995-P
Lot	HVN****	QBU****	XSM****
Gender	Male	Male	Female
Age	33	33	59
BMI	31.0	31.0	27.1
EBV	IgG+	IgG+	IgG+
RPR	Neg	Neg	Neg
CMV	Neg	Neg	Pos
COD	Anoxia 2nd to Natural Causes/Cardiovascular	Anoxia 2nd to Natural Causes/Cardiovascular	CVA
General	Depression, right wrist and ligament repair s/p MVC 7 yrs ago.	Depression, right wrist and ligament repair s/p MVC 7 yrs ago.	Urethra surgery (short urethra w/ frequent UTIs).
Meds	Celexa, Seroquel	Celexa, Seroquel	None
Alcohol	Beer:1-2 times/week, 6 pack on weekends, 3-4 on weekdays for 12 years	Beer:1-2 times/week, 6 pack on weekends, 3-4 on weekdays for 12 years	None reported
Tobacco	0.5-1 ppd cigarettes × 15 yrs, current	0.5-1 ppd cigarettes × 15 yrs, current	1 ppd x 40 yrs
Drug	Meth for 6 mos, used 1-2 times/week, last used 7 years ago, IVDA 7 yrs ago	Meth for 6 mos, used 1-2 times/week, last used 7 years ago, IVDA 7 yrs ago	None reported

HPH represents human primary hepatocytes

Supplementary Table 2. Antibodies used for western blot, immunofluorescence (IF) and immunohistochemistry (IHC) staining

Antibody	Company	Catalogue No.	Reactivity	Application used in this study	Observed Molecular weight (kDa)
GAPDH	Diagbio	db106	H, M	Western blot	37
HA tag	Diagbio	db2603	H, M	Western blot	N/A
FLAG tag	Diagbio	db7002	H, M	Western blot	N/A
P53	Santa Cruz Biotechnology	sc-126	H, M	Western blot; IF	53
P53	Origene	TA502870	H, M	IHC staining	N/A
ERK	Santa Cruz Biotechnology	sc-135900	H, M	Western blot	42
phospho-ERK (Tyr204)	Santa Cruz Biotechnology	sc-7383	H, M	Western blot; IHC staining; IF	42
EphA2	Cell Signaling Technology	#6997	H, M	Western blot; IF	130
phospho-EphA2 (Ser897)	Cell Signaling Technology	#6347	H, M	Western blot; IF	130

phospho-MDM2 (Ser166)	Cell Signaling Technology	#3521	H, M	Western blot; IHC staining	90
LC3A/B	Cell Signaling Technology	#4108	H, M	Western blot	14, 16
phospho-Akt (Ser473)	Cell Signaling Technology	#4060	H, M	Western blot	65
phospho-EphA2 (Tyr588)	Cell Signaling Technology	#12677	H, M	Western blot	130
MDM2	Huabio	RT1382	H,M	Western blot	55, 90
Cleaved PARP	Huabio	ET1608-10	H	Western blot	89
Lamin B1	Huabio	R1508-1	H, M	Western blot	66
Cleaved PARP	Abcam	ab32064	M	Western blot	25
MDM2	ABclonal	A13327	H,M	Western blot	55
EFNA1	ABclonal	A9132	H, M	Western blot	25
MDM2	Affinity Biosciences LTD	AF0208	H, M	IF	N/A

H represents human; M represents mouse

Supplementary Table 3. Comparison table of figure 17c drug screening

	Name	CAS	p-EphA2(Ser897)/ EphA2
F1	Lisinopril dihydrate	83915-83-7	1.09
F2	Carbaglu	1188-38-1	1.48
F3	Pyridoxine hydrochloride	58-56-0	1.61
F4	Acetohexamide	968-81-0	1.77
F5	Guaifenesin	93-14-1	1.70
F6	L(+)-Ascorbic acid	50-81-7	1.82
F7	Dicyclomine hydrochloride	67-92-5	1.66
F8	Chlorpheniramine maleate	113-92-8	0.97
F9	Pentoxifylline	6493-05-6	0.22
F10	Flavoxate hydrochloride	3717-88-2	2.51
F11	Disodium monofluorophosphate	10163-15-2	1.74
F12	Mepenzolate Bromide	76-90-4	1.13
F13	Diflunisal	22494-42-4	0.29
F14	(R)-Naproxen	23979-41-1	1.22
F15	Panthenol	16485-10-2	1.07
F16	Fexofenadine hydrochloride	138452-21-8	1.93
F17	Aripiprazole	129722-12-9	7.54
F18	Clofibric acid	882-09-7	2.11
F19	Prasugrel	150322-43-3	0.30
F20	Rosiglitazone	122320-73-4	0.18
F21	Metoprolol tartrate	56392-17-7	0.18

F22	Acebutolol hydrochloride	34381-68-5	0.20
F23	Perindopril erbumine	107133-36-8	0.59
F24	Nifedipine	21829-25-4	0.82
F25	Flavin mononucleotide	130-40-5	1.65
F26	Nicardipine hydrochloride	54527-84-3	1.19
F27	Phenylephrine hydrochloride	61-76-7	0.98
F28	Telmisartan	144701-48-4	1.00
F29	Irbesartan	138402-11-6	0.65
F30	Fludrocortisone acetate	514-36-3	0.77
F31	Glipizide	29094-61-9	0.37
F32	Vorapaxar sulfate	705260-08-8	0.49
F33	Methazolamide	554-57-4	1.02
F34	Dapagliflozin	461432-26-8	1.69
F35	Fluocinonide	356-12-7	0.82
F36	Bumetanide	28395-03-1	0.56
F37	Rizatriptan benzoate	145202-66-0	1.21
F38	Levetiracetam	102767-28-2	1.22
F39	Anagrelide	68475-42-3	1.10
F40	Fluocinonide	356-12-7	1.23
F41	Bumetanide	28395-03-1	1.16
N1	Prim-O-glucosylcimifugin	80681-45-4	0.70
N2	Crocin I	42553-65-1	0.34
N3	Ligustroflavone	260413-62-5	0.24
N4	Daidzein	486-66-8	0.20

N5	Saikosaponin C	20736-08-7	0.23
N6	Isoimperatorin	482-45-1	0.43
N7	Ginsenoside CK	39262-14-1	0.77
N8	Glabridin	59870-68-7	0.88
N9	2,3,5,4'-tetrahydroxyl diphenylethylene-2-o-glucoside	82373-94-2	0.59
N10	Schisantherin A	58546-56-8	2.08
N11	Isoalantolactone	470-17-7	2.41
N12	Tyrosol	501-94-0	1.89
N13	Diosgenin glucoside	14144-06-0	0.93
N14	Shikonin	517-89-5	0.92
N15	Schizandrin B	61281-37-6	0.75
N16	Quercitrin	522-12-3	0.71
N17	Catalpol	2415-24-9	0.60
N18	Ginkgolide A	15291-75-5	0.61
N19	Costundide	553-21-9	0.70
N20	Scutellarein	529-53-3	1.61
N21	(−)-Epicatechin gallate	1257-08-5	0.99
N22	Orientin	28608-75-5	0.93
N23	Schizandrin B	61281-37-6	0.99
N24	Daidzin	552-66-9	0.84
N25	Calycosin	20575-57-9	0.91
N26	Glycitein	40957-83-3	0.68
N27	Berberine	2086-83-1	0.51

N28	Isoquercitrin	482-35-9	2.28
N29	Curcumo	4871-97-0	1.45
N30	Astragalin	480-10-4	1.00
N31	Wogonin	632-85-9	1.10
N32	Dehydrocostus Lactone	477-43-0	2.19
N33	Tectoridin	611-40-5	0.78
N34	Peimine	23496-41-5	0.55
N35	Psoralen	66-97-7	0.67
N36	(+)-Catechin hydrate	225937-10-0	0.44
N37	Schisandrin A	61281-38-7	0.72
N38	Schisandrol B	58546-54-6	0.87
N39	Schisandrin C	61301-33-5	3.45
N40	Schisantherin D	64917-82-4	0.78
N41	Echinacoside	82854-37-3	0.23
N42	Dioscin	19057-60-4	7.12
N43	3-Hydroxy-4-methoxycinnamic acid	537-73-5	2.00
N44	Syringin	118-34-3	1.93
N45	Bisdemethoxycurcumin	24939-16-0	2.96

Supplementary Table 4. The primer sequences of quantitative real time polymerase chain reaction

Gene	Forward	Reverse
H-*ACTB*	CACCATTGGCAATGAGCGGTTC	AGGTCTTTGCGGATGTCCACGT
H-*TP53*	ATCTACAAGCAGTCACAG	TCATCCAAATACTCCACACGC
M-*Actb*	GTGACGTTGACATCCGTAAAGA	GCCGGACTCATCGTACTCC
M-*Trp53*	ATGTTCCGGGAGCTGAATG	CCCCACTTTCTTGACCATTG

H represents human; M represents mouse

Supplementary Table 5. Oligonucleotide sequences of siRNAs

**Name**	**Anti-sense sequences**
si*EphA2* #1	5'-GACAGACAUAUAGGAUAUUdTdT-3´
si*EphA2* #2	5'-AUCAAGAUGCAGCAGUAUAdTdT-3´
si*TP53* #1	5'-AGACCUAUGGAAACUACUUdTdT-3´
si*TP53* #2	5'-CCAUCCACUACAACUACAUdTdT-3´
si*EFNA1* #1	5'-CGUGUAUAGUAUCUGUAUAdTdT-3´
si*EFNA1* #2	5'-GGUGCGGUCUAGUGAUCUAdTdT-3´
si*DDR2*	5'-UGGCUUGGUGUCUUACAAUdTdT-3´
si*TRKA*	5'-UCUACAGCACCGACUAUUAdTdT-3´
si*SAPK2*	5'-GAAUCUACACGCAUGUAUGdTdT-3´
si*PTK5*	5'-AUUUGAUUUGUCGUAUAAAdTdT-3´
