# Supplementary material for: Regorafenib inhibits EphA2 phosphorylation and leads to liver damage via the ERK/MDM2/p53 axis
Source: Nat Commun. 2023 May 13;14:2756. doi: 10.1038/s41467-023-38430-8 (PMC10182995; doi:10.1038/s41467-023-38430-8)
Supplement: Supplementary file 3 — Reporting Summary [file 41467_2023_38430_MOESM3_ESM.pdf]

nature portfolio

Corresponding author(s): Peihua Luo

Last updated by author(s): Apr 28, 2023

Reporting Summary

Nature Portfolio wishes to improve the reproducibility of the work that we publish. This form provides structure for consistency and transparency in reporting. For further information on Nature Portfolio policies, see our Editorial Policies and the Editorial Policy Checklist.

Statistics

For all statistical analyses, confirm that the following items are present in the figure legend, table legend, main text, or Methods section.

n/a	Confirmed	
☐	☒	The exact sample size (*n*) for each experimental group/condition, given as a discrete number and unit of measurement
☐	☒	A statement on whether measurements were taken from distinct samples or whether the same sample was measured repeatedly
☐	☒	The statistical test(s) used AND whether they are one- or two-sided<br>*Only common tests should be described solely by name; describe more complex techniques in the Methods section.*
☒	☐	A description of all covariates tested
☒	☐	A description of any assumptions or corrections, such as tests of normality and adjustment for multiple comparisons
☐	☒	A full description of the statistical parameters including central tendency (e.g. means) or other basic estimates (e.g. regression coefficient) AND variation (e.g. standard deviation) or associated estimates of uncertainty (e.g. confidence intervals)
☐	☒	For null hypothesis testing, the test statistic (e.g. *F*, *t*, *r*) with confidence intervals, effect sizes, degrees of freedom and *P* value noted<br>*Give P values as exact values whenever suitable.*
☒	☐	For Bayesian analysis, information on the choice of priors and Markov chain Monte Carlo settings
☒	☐	For hierarchical and complex designs, identification of the appropriate level for tests and full reporting of outcomes
☒	☐	Estimates of effect sizes (e.g. Cohen's *d*, Pearson's *r*), indicating how they were calculated

Our web collection on statistics for biologists contains articles on many of the points above.

Software and code

Policy information about availability of computer code

Data collection	The absorbance of SRB (Sulforhodamine B) colorimetry was measured using a multiscan spectrum (Thermo Fisher Scientific, Marietta, Ohio, USA). qRT-PCR was performed by using iTaq Universal SYBR Green Supermix (1725125, Bio-Rad, CA, USA) in a CFX96TM Real-Time System (Bio-Rad, CA, USA). The histological images were observed and captured under Aperio AT2 (Leica Biosystems, Heidelberg, Germany). Western blot was carried out using Western Lightning Plus-ECL reagent (NEL105001EA, PerkinElmer, Waltham, MA, USA) and membranes were exposed using Amersham Imager 600 (General Electric Company, Boston, MA, USA). Flow cytometry was performed using FACS-Calibur cytometer (BD Biosciences, New Jersey, USA) and recorded by BD CellQuest Pro software (version 5.1).
Data analysis	Microsoft Excel (Version 1808)<br>Graph pad Prism (version 8.0)<br>Image J (version 1.8.0).

For manuscripts utilizing custom algorithms or software that are central to the research but not yet described in published literature, software must be made available to editors and reviewers. We strongly encourage code deposition in a community repository (e.g. GitHub). See the Nature Portfolio guidelines for submitting code & software for further information.

Data

Policy information about availability of data

All manuscripts must include a data availability statement. This statement should provide the following information, where applicable:

- Accession codes, unique identifiers, or web links for publicly available datasets
- A description of any restrictions on data availability
- For clinical datasets or third party data, please ensure that the statement adheres to our policy

The authors declare that the main data supporting the findings of this study are available in this Article, its Supplementary Information and Source Data. Extra data for the individual measurements are available on request. The source data underlying Fig. lb-e, 1g-i, 2a-f, 2h-l, 3b-e, 3g-j, 4b-d, 4f-h, 5a-d, 5f-h, 6a, 6c-f, 7b, 7d-e, 8a-b, 8d-f as well as Supplementary Fig. 1a, 1d, 2a-e, 3a-b, 3d, 4a-b, 5a-c, 7a, 8a-e, 9, 11a-c, 13a-d, 14b, 16a-b, 17a-d, 18a-b, 18d, 19b-c, 20, 21 are provided as a Source Data file. Source data are provided with this paper.

Research involving human participants, their data, or biological material

Policy information about studies with human participants or human data. See also policy information about sex, gender (identity/presentation), and sexual orientation and race, ethnicity and racism.

Reporting on sex and gender	n/a
Reporting on race, ethnicity, or other socially relevant groupings	n/a
Population characteristics	n/a
Recruitment	n/a
Ethics oversight	n/a

Note that full information on the approval of the study protocol must also be provided in the manuscript.

Field-specific reporting

Please select the one below that is the best fit for your research. If you are not sure, read the appropriate sections before making your selection.

☒ Life sciences ☐ Behavioural & social sciences ☐ Ecological, evolutionary & environmental sciences

For a reference copy of the document with all sections, see nature.com/documents/nr-reporting-summary-flat.pdf

Life sciences study design

All studies must disclose on these points even when the disclosure is negative.

Sample size	All the sample sizes were chosen based on our previous experience to obtain the magnitude and consistency of measurable differences between groups. Exact sample size are recorded in the paper.
Data exclusions	No data was excluded.
Replication	We confirm all attempts at replication were successful. The experimental findings were repeated and our data are based on at least three independent experiments with similar results unless otherwise noted in the manuscript. Details are described in the legends of the corresponding figures.
Randomization	All samples were randomly allocated into experimental groups.
Blinding	For animal study, the investigators were blinded for the animal allocation to the experimental groups. All data collection and analyses were performed in a blind manner. For in vitro study, blinding was not applicable because the investigator in charge of an experiment was responsible for cell culture, treatment, sample collection, and data analysis.

Reporting for specific materials, systems and methods

We require information from authors about some types of materials, experimental systems and methods used in many studies. Here, indicate whether each material, system or method listed is relevant to your study. If you are not sure if a list item applies to your research, read the appropriate section before selecting a response.

Materials & experimental systems

n/a	Involved in the study
	☒ Antibodies
	☒ Eukaryotic cell lines
☒	☐ Palaeontology and archaeology
	☒ Animals and other organisms
☒	☐ Clinical data
☒	☐ Dual use research of concern
☒	☐ Plants

Methods

n/a	Involved in the study
☒	☐ ChIP-seq
	☒ Flow cytometry
☒	☐ MRI-based neuroimaging

Antibodies

Antibodies used

Anti-GAPDH (rabbit polyclonal); Diagbio; Cat #db106; Lot# U1021P; 1:10000 for WB
Anti-HA tag (rabbit polyclonal); Diagbio; Cat #db2603; Lot# S0319C; 1:1000 for WB
Anti-FLAG tag (rabbit polyclonal); Diagbio; Cat #db7002; Lot# W0421P; 1:1000 for WB
Anti-p53 (mouse monoclonal, clone DO-1); Santa Cruz Biotechnology; Cat #sc-126; Lot# F2117; 1:1000 for WB, 1:100 for IF
Anti-p53 (mouse monoclonal, clone OTI5E2); Origene; Cat #TA502870; Lot# F001; 1:100 for IHC
Anti-ERK (mouse monoclonal, clone MK1); Santa Cruz Biotechnology; Cat #sc-135900; Lot# K1920; 1:1000 for WB
Anti-phospho-ERK (Tyr204) (mouse monoclonal, clone E-4); Santa Cruz Biotechnology; Cat #sc-7383; Lot# K1521; 1:1000 for WB, 1:100 for IHC, 1:200 for IF
Anti-EphA2 (rabbit monoclonal, clone D4A2); Cell Signaling Technology; Cat #6997; Lot# 1; 1:1000 for WB, 1:100 for IF
Anti-phospho-EphA2 (Ser897) (rabbit monoclonal, clone D9A1); Cell Signaling Technology; Cat #6347; Lot# 1; 1:1000 for WB, 1:100 for IF
Anti-phospho-MDM2 (Ser166) (rabbit polyclonal); Cell Signaling Technology; Cat #3521; Lot# 5; 1:1000 for WB, 1:150 for IHC
Anti-LC3A/B (rabbit polyclonal); Cell Signaling Technology; Cat #4108; Lot# 5; 1:1000 for WB
Anti-phospho-Akt (Ser473) (rabbit monoclonal, clone D9E); Cell Signaling Technology; Cat #4060; Lot# 25; 1:1000 for WB
Anti-phospho-EphA2 (Tyr588) (rabbit monoclonal, clone D7X2L); Cell Signaling Technology; Cat #12677; Lot# 1; 1:1000 for WB
Anti-MDM2 (mouse monoclonal, clone 3G2); Huabio; Cat #RT1382; Lot# C0620; 1:1000 for WB
Anti-cleaved-PARP (rabbit monoclonal, clone SU0314); Huabio; Cat #ET1608-10; Lot# HN1102; 1:1000 for WB
Anti-Lamin B1 (rabbit polyclonal); Huabio; Cat #R1508-1; Lot# H20723; 1:1000 for WB
Anti-MDM2 (rabbit polyclonal); ABclonal; Cat #A13327; Lot# 5500023575; 1:1000 for WB
Anti-EFNA1 (rabbit monoclonal, clone ARC1443); ABclonal; Cat #A9132; Lot# 4000001443; 1:1000 for WB
Anti-cleaved-PARP (rabbit monoclonal, clone E51); Abcam; Cat #ab32064; Lot# GR3292031-9; 1:1000 for WB
Anti-MDM2 (rabbit polyclonal); Affinity Biosciences LTD; Cat #AF0208; Lot# 86r9325; 1:200 for IF

Validation

Anti-GAPDH (Rabbit polyclonal)
Suitable for: WB, IHC, ICC/IF, FC
http://www.diagbio.com/product/PNOdb106.html

Anti-HA tag
Suitable for: WB, IHC, ICC/IF, FC, IP
http://www.diagbio.com/product/PNOdb2603.html

Anti-FLAG tag
Suitable for: WB, IHC, ICC/IF, FC
http://www.diagbio.com/product/PNOdb7002.html

Anti-p53
Suitable for: WB, IP, IF, IHC(P), FCM
https://www.scbt.com/p/p53-antibody-do-1?requestFrom=search

Anti-p53
Suitable for: FC, IF, IHC, WB
https://www.origene.com.cn/catalog/antibodies/primary-antibodies/ta502870/p53-tp53-mouse-monoclonal-antibody-clone-id-oti5e2

Anti-ERK
Suitable for: WB, IP, IF
https://www.scbt.com/p/erk-1-2-antibody-mk1?requestFrom=search

Anti-phospho-ERK (Tyr204)
Suitable for: IF, IHC(P), FCM
https://www.scbt.com/p/p-erk-antibody-e-4?requestFrom=search

Anti-EphA2

Suitable for: WB, IP, IF, IHC
https://www.cellsignal.cn/products/primary-antibodies/epha2-d4a2-xp-rabbit-mab/6997?site-search-type=Products&N=4294956287&Ntt=6997&fromPage=plp&_requestid=1945729

Anti-phospho-EphA2 (Ser897)
Suitable for: WB, IP, IHC
https://www.cellsignal.cn/products/primary-antibodies/phospho-epha2-ser897-d9a1-rabbit-mab/6347?site-search-type=Products&N=4294956287&Ntt=6347&fromPage=plp&_requestid=1946481

Anti-phospho-MDM2 (Ser166)
Suitable for: WB
https://www.cellsignal.cn/products/primary-antibodies/phospho-mdm2-ser166-antibody/3521?site-search-type=Products&N=4294956287&Ntt=3521&fromPage=plp

Anti-LC3A/B
Suitable for: WB, IF, F
https://www.cellsignal.cn/products/primary-antibodies/lc3a-b-antibody/4108?site-search-type=Products&N=4294956287&Ntt=4108&fromPage=plp&_requestid=1947378

Anti-phospho-Akt (Ser473)
Suitable for: WB, IP, IHC, IF, F
https://www.cellsignal.cn/products/primary-antibodies/phospho-akt-ser473-d9e-xp-rabbit-mab/4060?site-search-type=Products&N=4294956287&Ntt=4060&fromPage=plp&_requestid=1947666

Anti-phospho-EphA2 (Tyr588)
Suitable for: WB, IP
https://www.cellsignal.cn/products/primary-antibodies/phospho-epha2-tyr588-d7x2l-rabbit-mab/12677?site-search-type=Products&N=4294956287&Ntt=12677&fromPage=plp&_requestid=1951114

Anti-MDM2
Suitable for: WB,IP,IF,IHC-P
http://www.huabio.cn/product/MDM2-antibody-RT1382

Anti-cleaved-PARP
Suitable for: WB,IF-Cell,IP,FC
http://www.huabio.cn/product/Cleaved-PARP-antibody-ET1608-10

Anti-Lamin B1
Suitable for: WB
http://www.huabio.cn/product/Lamin-B1-antibody-R1508-1

Anti-MDM2
Suitable for: WB
https://abclonal.com.cn/catalog/A13327

Anti-EFNA1
Suitable for: WB, IHC, IF/ICC
https://abclonal.com.cn/catalog/A9132

Anti-cleaved-PARP
Suitable for: WB, IHC-P
https://www.abcam.cn/products/primary-antibodies/cleaved-parp1-antibody-e51-ab32064.html

Anti-MDM2
Suitable for: WB, IHC, IF
https://www.affbiotech.cn/goods-136-AF0208-MDM2_Antibody.html

Eukaryotic cell lines

Policy information about cell lines and Sex and Gender in Research

Cell line source(s)	The human primary hepatocytes were provided by BioreclamationIVT (F00995-P and M00995-P, Hicksville, New York, USA), and the clinical characteristics of the donors are shown in Table S1-S3. HL-7702 (JNO-048) was purchased from GuangZhou Jennio Biotech Co.,Ltd. SW-480 (TCHu 86), HCT-116 (TCHu 99), Bel-7402 (TCHu 10), Hep-G2 (TCHu 72), and HEK-293T (GNHu 18) cells were originally obtained from the Cell Bank of China Science Academy.
Authentication	All the cell lines were obtained from suppliers which comprehensively perform authentication and quality control tests on all cell lines and were used without modification once received from the suppliers.

Mycoplasma contamination	Cells lines used in this study tested negative for mycoplasma contamination.
Commonly misidentified lines (See ICLAC register)	No commonly misidentified cell lines were used in this study.

Animals and other research organisms

Policy information about studies involving animals; ARRIVE guidelines recommended for reporting animal research, and Sex and Gender in Research

Laboratory animals	Animal experiments involved male and female mice, of the C57BL/6J, aged 6 weeks.
Wild animals	The study did not involve the use of wild animals.
Reporting on sex	There was no sex bias in the animals used in this study.
Field-collected samples	The study did not involve the use of samples collected from the field.
Ethics oversight	All experiments were conducted in accordance with protocols approved by the Center for Drug Safety Evaluation and Research of Zhejiang University. All mice were bred according to the protocol of the Institutional Animal Care and Use Committee (IACUC).

Note that full information on the approval of the study protocol must also be provided in the manuscript.

Flow Cytometry

Plots

Confirm that:

- [x] The axis labels state the marker and fluorochrome used (e.g. CD4-FITC).
- [x] The axis scales are clearly visible. Include numbers along axes only for bottom left plot of group (a 'group' is an analysis of identical markers).
- [x] All plots are contour plots with outliers or pseudocolor plots.
- [x] A numerical value for number of cells or percentage (with statistics) is provided.

Methodology

Sample preparation	Flow cytometry analysis of Annexin V-PI staining:<br>The apoptotic ratio was measured with an Pharmingen™ FITC Annexin V Apoptosis Detection Kit I (556547, BD Biosciences, New Jersey, USA). Procedures were performed according to the instructions. Briefly, cells were treated for the indicated time, harvested and washed with PBS for binding and Annexin V-PI staining.<br>Flow cytometry analysis of JC-1 staining:<br>Mitochondrial membrane potential (MMP) was detected by JC-1 staining assays. After treatment with regorafenib for the indicated time, cells were collected by trypsinization and stained with JC-1 (5 μM; T4069, Sigma-Aldrich, Shanghai, China) for 30 min at 37°C avoiding light.
Instrument	BD FACSCalibur™ Flow Cytometer
Software	BD CellQuest Pro software (version 5.1)
Cell population abundance	10000 cells were analyzed in each sample.
Gating strategy	Forward and lateral scattering (FSC-A versus SSC-A) was used to identify the cell populations of interest. For flow cytometry analysis of Annexin V-PI staining, apoptotic cells were gated using appropriate channels by gating unstained cells and single stained cells. For flow cytometry analysis of JC-1 staining, the mitochondrial membrane potential depolarized cells were gated using appropriate channels by gating positive cells treated with CCCP (10 μM; C6700, Solarbio, Beijing, China) for 20 min.

- [x] Tick this box to confirm that a figure exemplifying the gating strategy is provided in the Supplementary Information.
